# Supplementary figures and images for: Fibroblast-induced mammary epithelial branching depends on fibroblast contractility
Source: PLoS Biol. 2024 Jan 10;22(1):e3002093. doi: 10.1371/journal.pbio.3002093 (PMC10805323; doi:10.1371/journal.pbio.3002093)

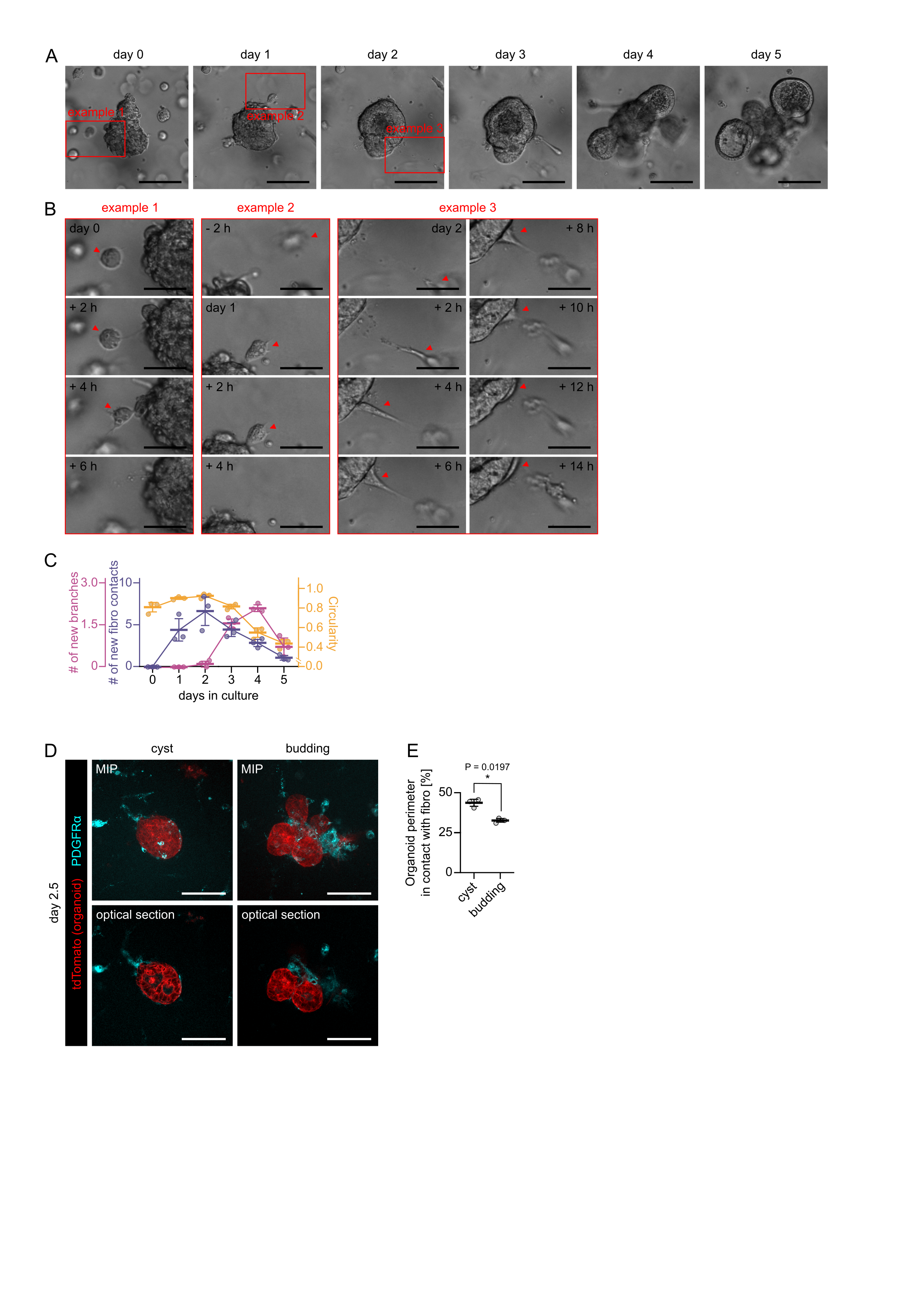

Supplement: S2 Fig — (A) Time-lapse snapshots of an organoid-fibroblast co-culture. Scale bar: 100 μm. (B) Detailed snapshots of 3 examples of fibroblast-organoid contact establishment in the co-cultures shown in (A) on days 1, 2, and 3. Red arrowheads indicate fibroblasts of interest. Scale bar: 50 μm. (C) Quantification of organoid circularity (data from Fig 1), number of new branches and number of established fibroblast-organoid contacts from matched experiments. The plot shows mean ± SD; n = 3 (each dot represents the average from a biologically independent experiment, N = 20 organoids per experiment). (D) Maximum intensity projection (MIP) and optical section images of a dispersed co-culture on day 2.5, representative images of cystic and budding organoids (tdTomato). Fibroblasts were detected by immunostaining for PDGFRα. Scale bar: 100 μm. (E) Quantification of organoid middle section perimeter in contact with PDGFRα signal. The plot shows mean ± SD. Each dot represents an average from 1 experiment. Statistical analysis: two-tailored t test; n = 3 independent biological samples, N = 15–24 organoids per sample. The data underlying the graphs shown in the figure can be found in S1 Data. (TIFF) [file pbio.3002093.s002.tiff]

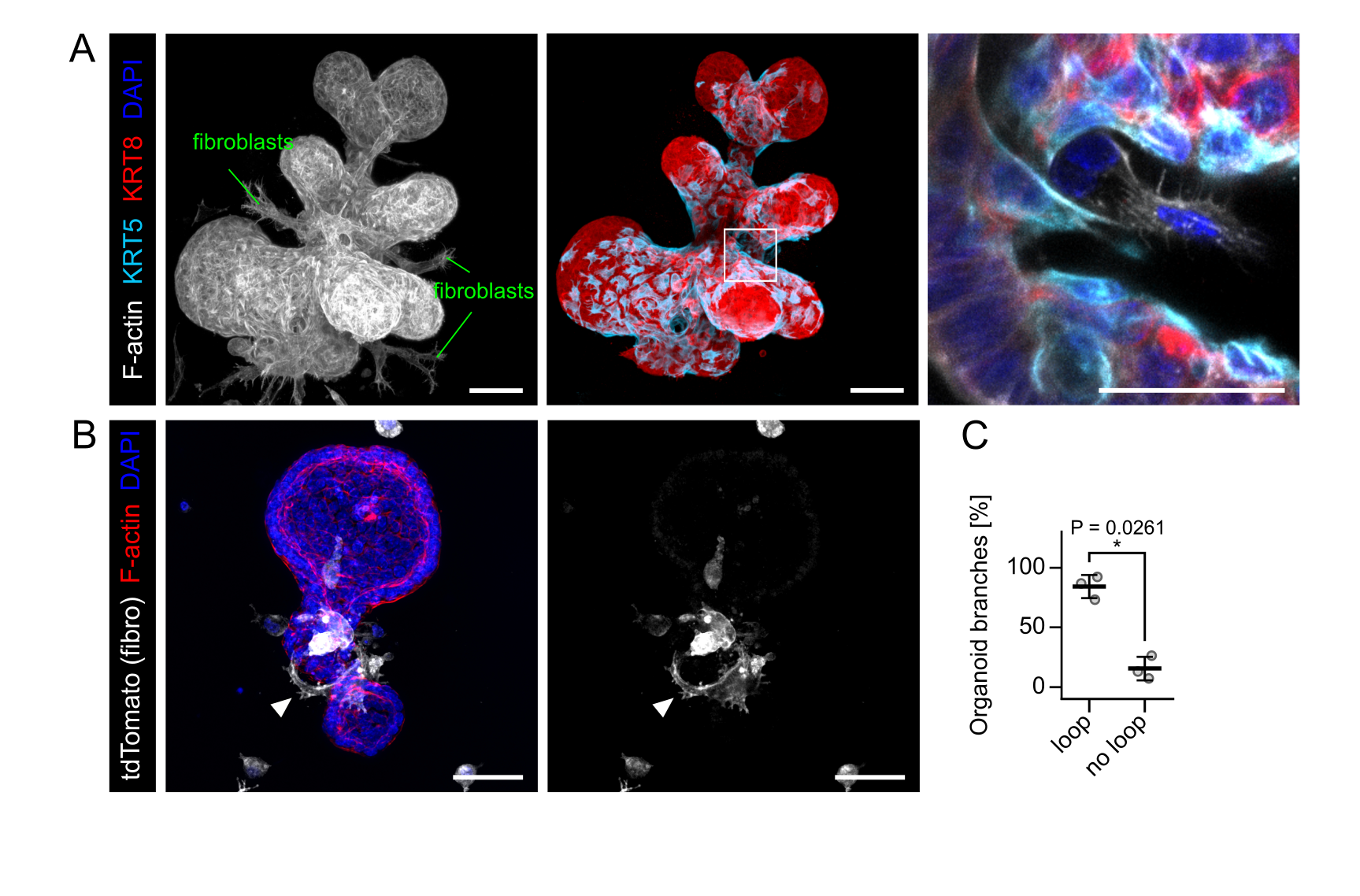

Supplement: S3 Fig — (A) A representative confocal image of a dispersed co-culture on day 4. Scale bar: 20 μm, scale bar in detail: 10 μm. (B) A representative confocal image of a dispersed organoid-fibroblast co-culture on day 3. The arrowhead indicates the fibroblast loop at the branch neck. Scale bar: 50 μm. (C) Quantification of the presence of fibroblast loops around organoid branches in dispersed co-cultures. The plot shows mean ± SD. Statistical analysis: two-tailored t test; n = 3 independent biological replicates, N = 5–12 organoids per experiment; 56 branches in total. The data underlying the graphs shown in the figure can be found in S1 Data. (TIFF) [file pbio.3002093.s003.tiff]

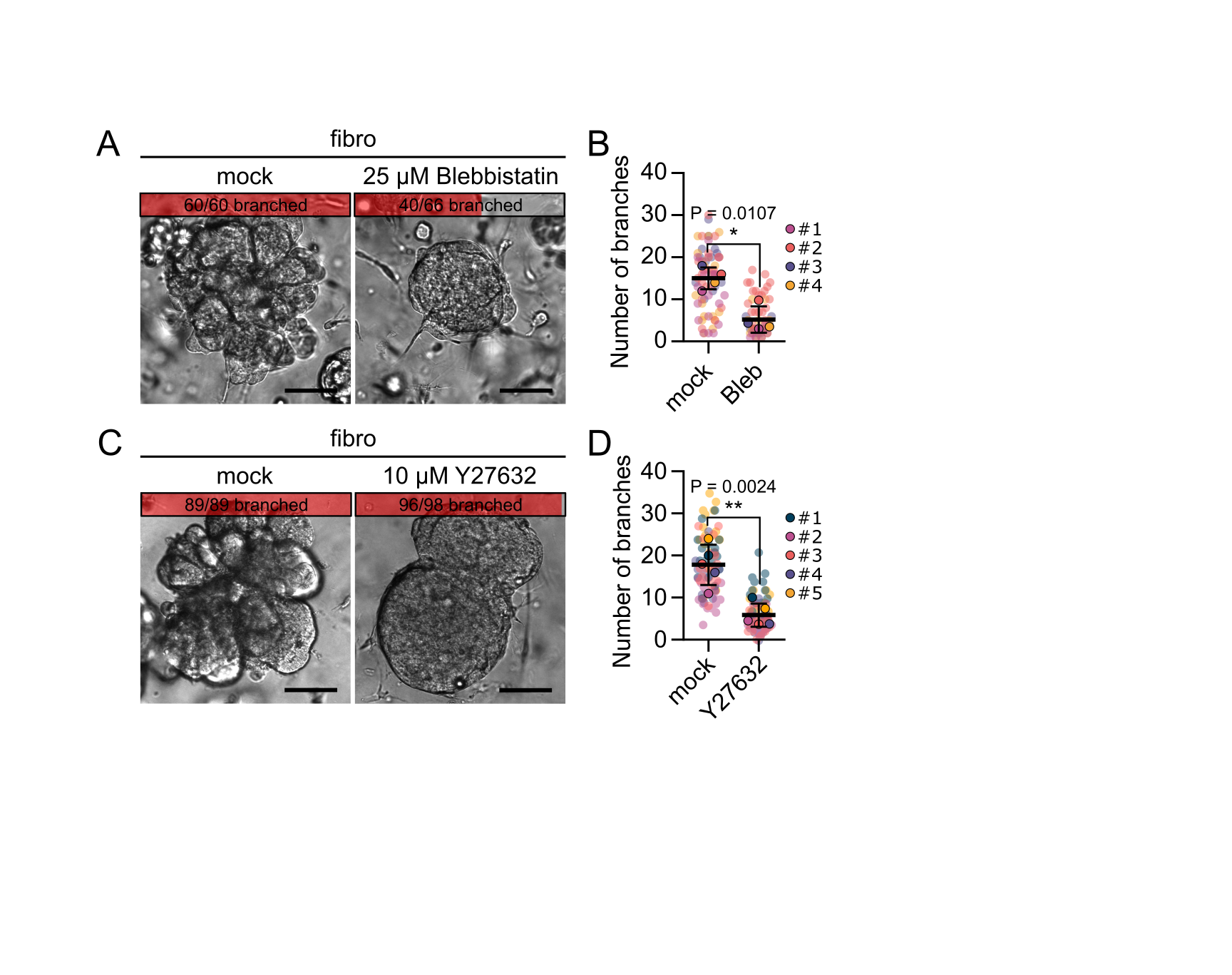

Supplement: S4 Fig — (A, C) Photographs of spheroids on day 4 of dispersed co-culture with fibroblasts upon treatment with no inhibitor (mock), with blebbistatin (Bleb, A) or with Y27632 (C). Top gray and red bars indicate proportion of branched spheroids out of all spheroids per condition. Scale bar: 100 μm. (B, D) Quantification of number of branches/buds per branched spheroid in conditions from (A). The plot shows mean ± SD, each lined dot shows mean from each experiment, each faint dot shows single spheroid measurement, n = 4 (B) or 5 (D) biologically independent experiments, N = 20 spheroids per experiment. Statistical analysis: two-tailored t test. The data underlying the graphs shown in the figure can be found in S1 Data. (TIFF) [file pbio.3002093.s004.tiff]

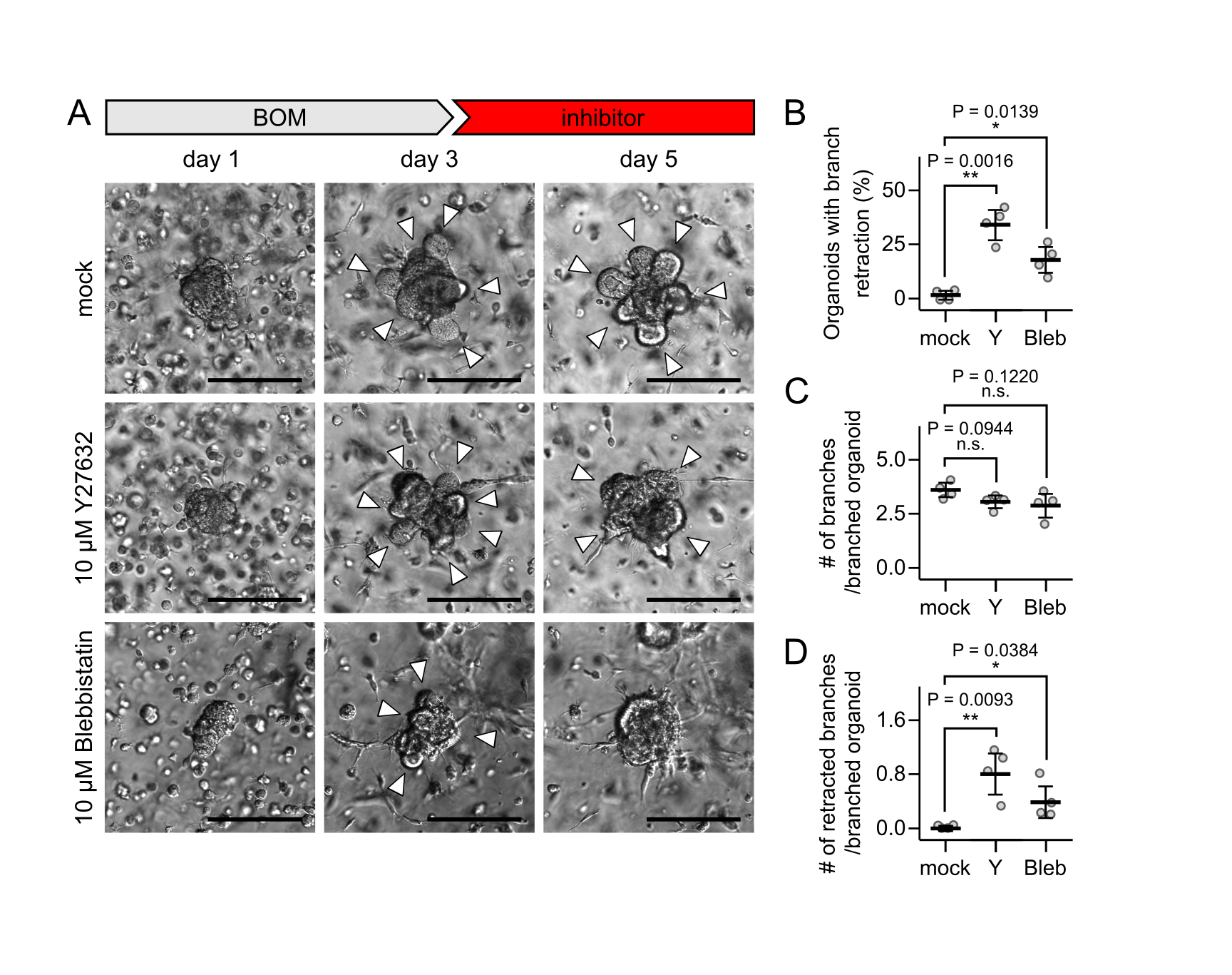

Supplement: S6 Fig — (A) Experimental scheme (top) and time-lapse snapshots of dispersed co-cultures treated with contractility inhibitors on day 3 of culture. Scale bar: 100 μm. White arrowheads indicate organoid branches. (B–D) Quantification of organoids with retracted branches (B), number of formed branches per branched organoids (C) and number of retracted branches per organoid (D). The plots show mean ± SD. Statistical analysis: two-tailored t test; n = 4 independent biological replicates, N = 20 organoids per experiment. The data underlying the graphs shown in the figure can be found in S1 Data. (TIFF) [file pbio.3002093.s006.tiff]

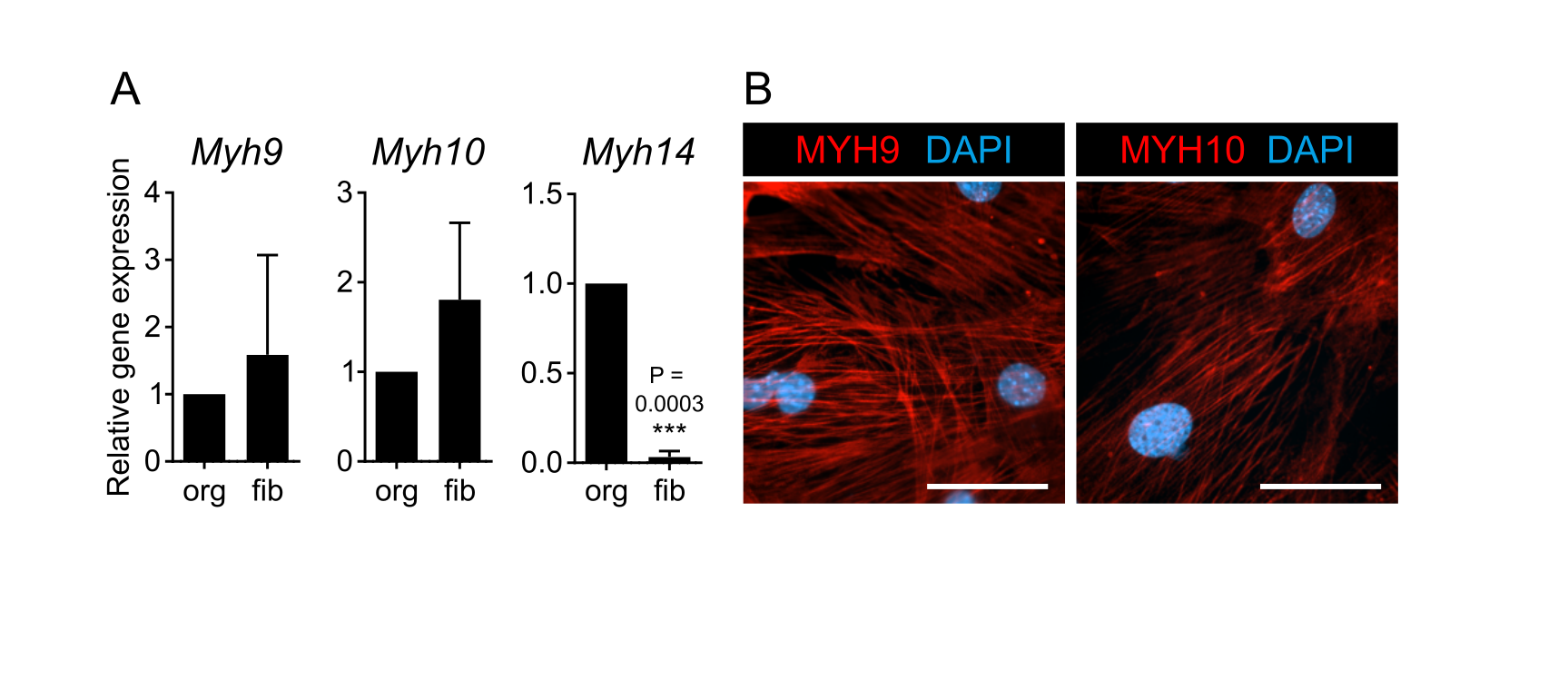

Supplement: S7 Fig — (A) Real-time qPCR analysis of non-muscle myosin II heavy chain genes Myh9, Myh10, and Myh14 in mammary fibroblasts (fib) and epithelium (organoids, org). Plots show mean ± SD. Statistical analysis: two-tailored t test; n = 3 independent biological samples. (B) Representative images of MYH9 and MYH10 immunostaining in mammary fibroblasts in the first passage. Scale bar: 50 μm. The data underlying the graphs shown in the figure can be found in S1 Data. (TIFF) [file pbio.3002093.s007.tiff]

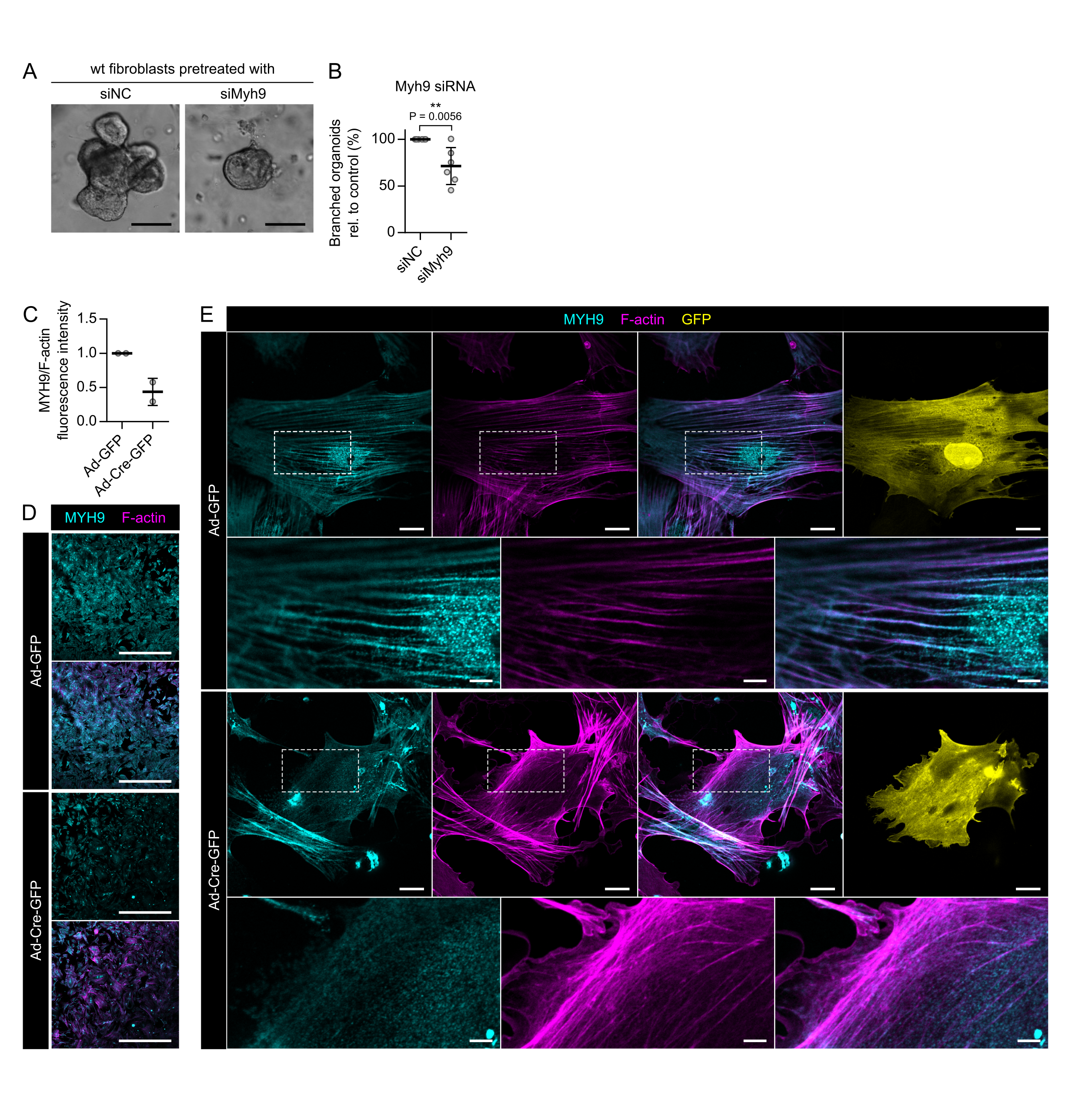

Supplement: S8 Fig — (A, B) Representative images (day 5 of culture) (A) and quantification (B) of organoid branching in dispersed co-cultures with wild-type fibroblasts pre-treated with nonsense (siNC) or Myh9 targeting (siMyh9) siRNA. Plot indicates mean ± SD. Statistical analysis: two-tailored paired t test; n = 6 independent Myh9 knockdown experiments; N = 20 organoids per each treatment of each independent experiment. Videos from the 5-day experiment are presented in S4 Movie. (C–E) Quantification of MYH9 down-regulation in Myh9 KO fibroblasts by immunofluorescence. The plot (C) shows mean ± SD, n = 2 independent experiments. Representative images (D) show MYH9 (cyan) and F-actin (phalloidin, magenta) staining in cultured primary mammary fibroblasts from Myh9fl/fl mice, treated with adeno-GFP (Ad-GFP) or adeno-Cre-GFP (Ad-Cre-GFP) vector, including details (E) of cytoskeleton organization. Scale bars: 1 mm (D), 20 μm (E, first and third row), and 5 μm (E, second and fourth row). The data underlying the graphs shown in the figure can be found in S1 Data. (TIFF) [file pbio.3002093.s008.tiff]

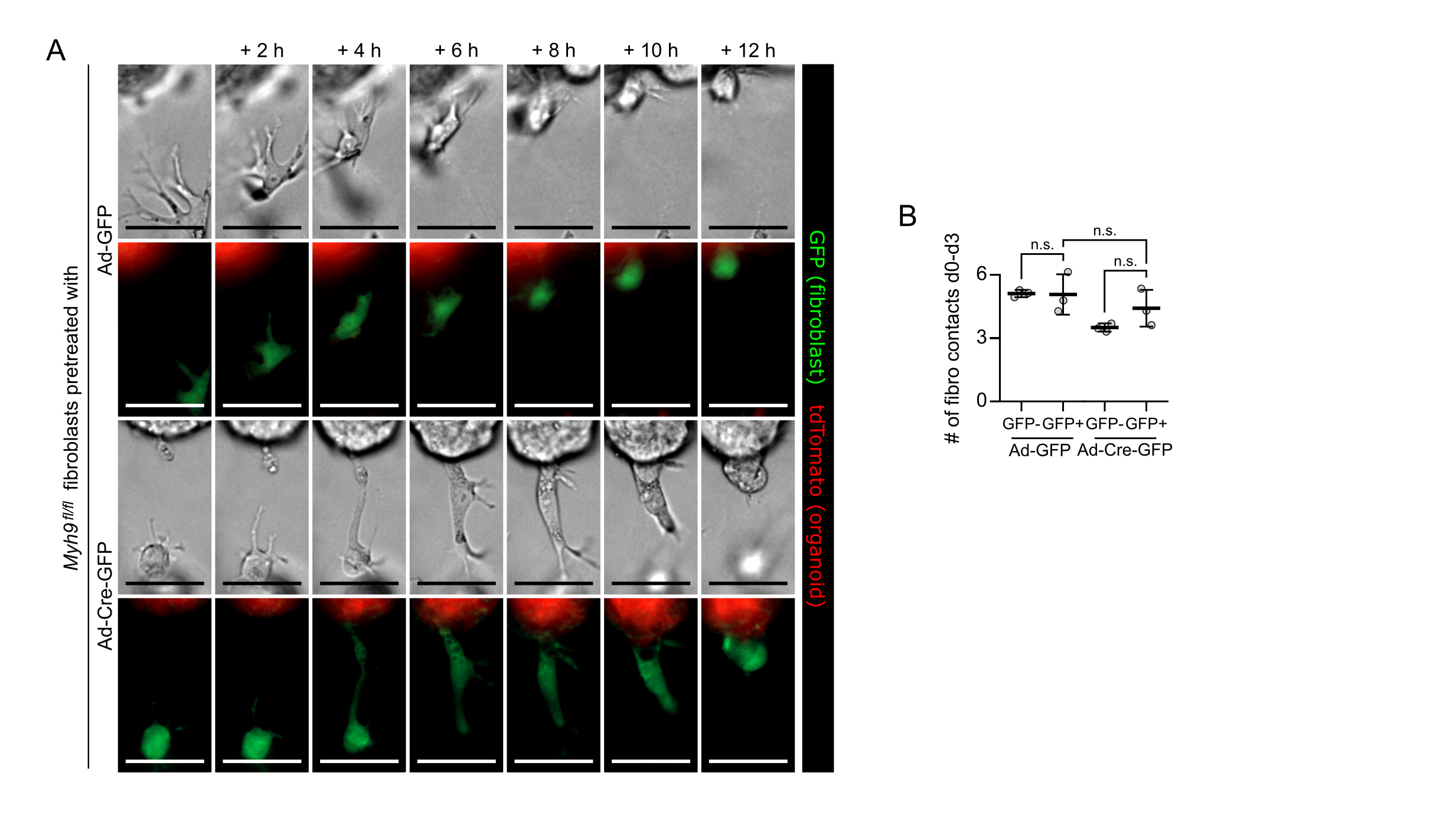

Supplement: S9 Fig — (A) Detailed time-lapse snapshots of fibroblast-organoid contact establishment in dispersed co-cultures with control or Myh9-KO fibroblasts and tdTomato+ organoids. Scale bar: 50 μm. (B) Quantification of fibroblast-organoid contacts established in the first 3 days of co-culture, comparing GFP+ and GFP- fibroblasts (GFP is a marker of adenoviral transduction). The plot shows mean ± SD. Statistical analysis: two-tailored t test; n = 3 independent biological replicates, N = 20 organoids per experiment. The data underlying the graphs shown in the figure can be found in S1 Data. (TIFF) [file pbio.3002093.s009.tiff]

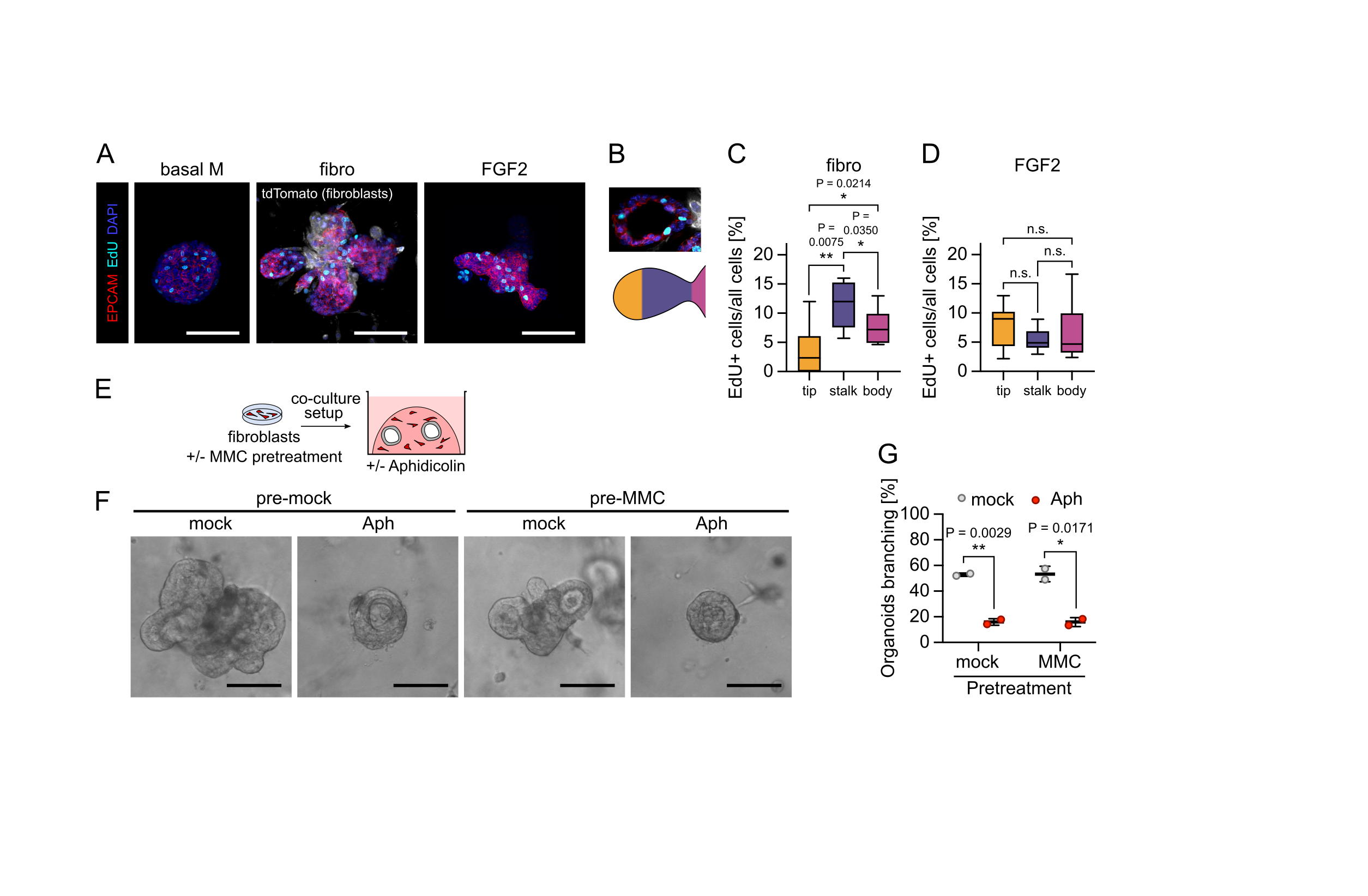

Supplement: S10 Fig — (A) Representative images of organoids on day 4 of culture in basal medium (basal M), in dispersed co-culture with fibroblasts or with FGF2, EdU administered 2 h pre-fix, EPCAM (red), DAPI (blue), EdU (cyan), fibroblasts were isolated from R26-mT/mG mice (tdTomato, white). Scale bar: 100 μm. (B) Optical section of a branch from (A) (top), a scheme of branch regions (bottom). (C, D) Quantification of percentage of EdU+ cells from epithelial cells in different branch regions in fibroblast-organoid dispersed co-culture (C) and in FGF2-treated organoid (D). The box and whiskers plot shows minimum, median, and maximum values, and second and third quartiles of data distribution. n = 3 independent experiments, N = 6 organoids, 2,202 analyzed cells in (C); N = 11 organoids, 3,104 analyzed cells in (D). Statistical analysis: Multiple t tests. (E) A scheme of the proliferation-inhibition experiment. (F) Co-cultures at day 5 (dispersed culture), fibroblasts pretreated with +/- mitomycin C (MMC), co-cultures treated with +/- aphidicolin (Aph). Scale bar: 100 μm. (G) Quantification of the percentage of branched organoids from experiment in (F). The plot shows mean ± SD, each dot represents a biologically independent experiment, n = 2, N = 51–77 organoids per sample, statistical analysis: t test. The data underlying the graphs shown in the figure can be found in S1 Data. (TIFF) [file pbio.3002093.s010.tiff]

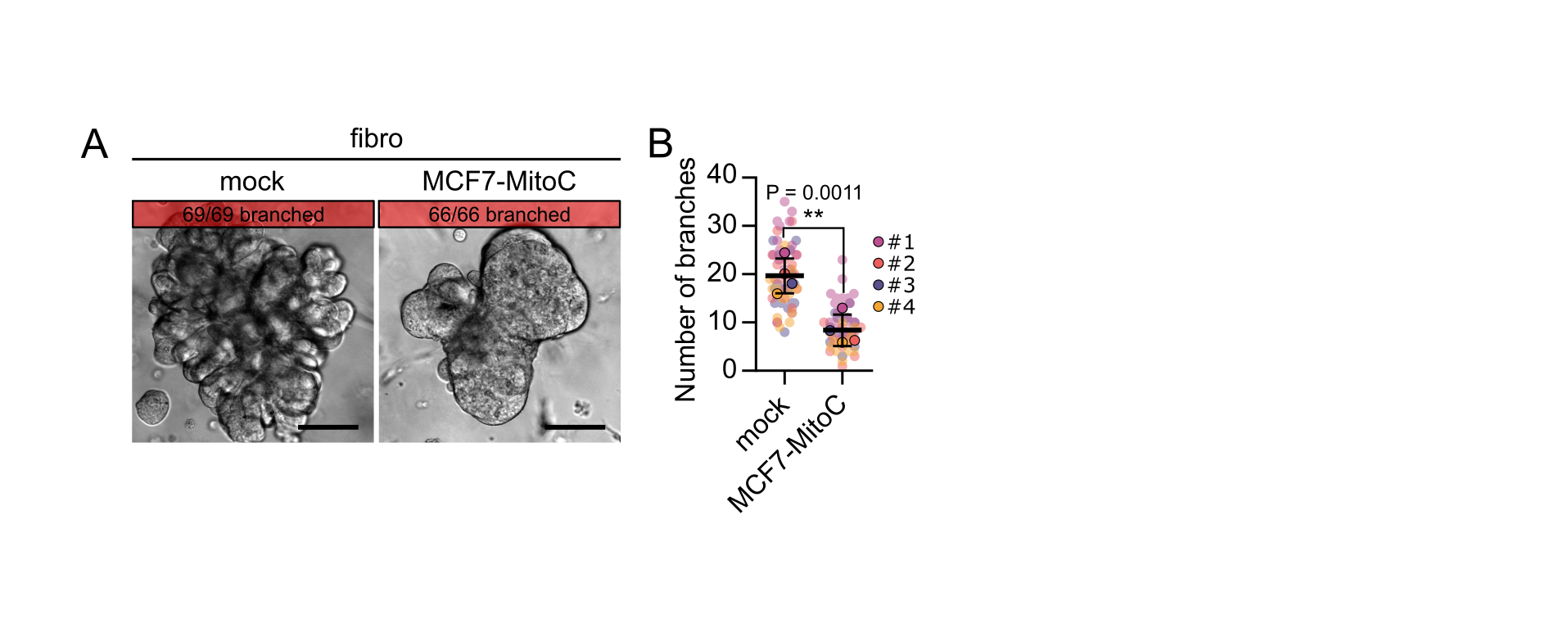

Supplement: S11 Fig — (A) Representative images of MCF7-ras spheroids in dispersed co-culture with fibroblasts on day 4 with spheroids formed from mock- or mitomycin C-treated MCF7-ras cells. The insets (top red bars) show proportion of branched spheroids out of all spheroids per condition. Scale bar: 100 μm. (B) The plot shows number of spheroid branches/buds formed, with mean ± SD. Each lined dot represents mean of each experiment, each faint dot represents 1 spheroid, n = 4 independent experiments (coded by dot colors), N = 15–20 spheroids per experiment. Statistical analysis: two-tailored t test. The data underlying the graphs shown in the figure can be found in S1 Data. (TIFF) [file pbio.3002093.s011.tiff]
