# Supplementary material for: Fibroblast-induced mammary epithelial branching depends on fibroblast contractility
Source: PLoS Biol. 2024 Jan 10;22(1):e3002093. doi: 10.1371/journal.pbio.3002093 (PMC10805323; doi:10.1371/journal.pbio.3002093)
Supplement: S1 Table — (DOCX) [file pbio.3002093.s020.docx]

| **Supplementary Table 1. The list of pharmacological and viral compounds.** | | | | |  |
| --- | --- | --- | --- | --- | --- |
| **Compound** | **Supplier** | **Cat. Number** | **Concentration used** |  |  |
| Aphidicolin | Merck | A4487 | 1.5 µM |  |  |
| Blebbistatin | Merck | B0560 | 10 μM |  |  |
| Mitomycin C | Merck | M4287 | 10 μg/ml |  |  |
| SU5402 | Merck | SML0443 | 1 μM, 5 μM, 10 μM |  |  |
| U0126 | Bio-techne | 1144 | 0.5 μM, 1 μM, 2 μM |  |  |
| Y27632 | Merck | SCM075; Y0503 | 10 μM |  |  |
| 4-OH tamoxifen | Sigma | H7904 | 0.5 mM |  |  |
| Adeno-GFP | Vector Biolabs | 1060 | 200 MOI |  |  |
| Adeno-Cre-GFP | Vector Biolabs | 1700 | 200 MOI |  |  |
| rAVCMV-LifeAct-TagGFP2 | IBIDI | 60120 | 500 MOI |  |  |
