# Supplementary material for: Fibroblast-induced mammary epithelial branching depends on fibroblast contractility
Source: PLoS Biol. 2024 Jan 10;22(1):e3002093. doi: 10.1371/journal.pbio.3002093 (PMC10805323; doi:10.1371/journal.pbio.3002093)
Supplement: S2 Table — (DOCX) [file pbio.3002093.s021.docx]

**Supplementary Table 2. The list of detection agents used in this study.**

| **Antibody** | **Host, class** | **Supplier** | **Cat. Number** | **Dilution** |
| --- | --- | --- | --- | --- |
| **ICC-IF** |  |  |  |  |
| MYH9 | Rabbit, polyclonal | Biolegend | 909801 | 1/1000 |
| MYH10 | Rabbit, polyclonal | Biolegend | 909901 | 1/1000 |
| *Secondary antibodies* |  |  |  |  |
| AlexaFluor 488 conjugated | Goat, polyclonal | Thermo Fisher Scientific | A-11001  A-11008 | 1/1000  1/1000 |
| Alexa Fluor 546 conjugated | Goat, polyclonal | Thermo Fisher Scientific | A-21133 | 1/1000 |
| Alexa Fluor 568 conjugated | Goat, polyclonal | Thermo Fisher Scientific | A-11011 | 1/1000 |
| Alexa Fluor 647 conjugated | Goat, polyclonal | Thermo Fisher Scientific | A-21235  A-21244 | 1/1000  1/1000 |
| **Organoid 3D staining** |  |  |  |  |
| Keratin 5 | Rabbit, polyclonal | Biolegend | 905504 | 1/250 |
| Keratin 8 | Rabbit, polyclonal | Biolegend | 904804 | 1/250 |
| KI67 | Rabbit, polyclonal | Zytomed | RBK027 | 1/300 |
| P-MYL9 (S19) | Mouse, monoclonal | Thermo Fisher Scientific | MA515163 | 1/100 |
| PDGFRα | Rabbit, monoclonal | Cell Signaling Technology | #3174 | 1/100 |
| P-ERK1/2 (T202/Y204) | Rabbit, monoclonal | Cell Signaling Technology | #4370 | 1/250 |
| YAP | Mouse, monoclonal | Santa Cruz Biotechnology | sc-101199 | 1/100 |
| Laminin α5 | Rabbit, polyclonal | (Rousselle and Aumailley, 1994) | N/A | 1/100 |
| αSMA | Mouse, monoclonal | Novus | NBP2-33006 | 1/400 |
| Vimentin | Chicken, polyclonal | Invitrogen | #PA1-16759 | 1/400 |
| EPCAM-AF647 | Rat, monoclonal | Biolegend | 118212 | 1/100 |
| *Secondary antibodies* as above | |  |  | 1/800 |
| Phalloidin-AlexaFluor488 | N/A | Thermo Fisher Scientific | A12379 | 1/200 |
| **Whole-gland staining** |  |  |  |  |
| Vimentin | Rabbit, monoclonal | Cell Signaling Technology | #5741 | 1/200 |
| *Secondary antibodies* as above |  | Thermo Fisher Scientific |  | 1/500 |
| **IHC-IF** |  |  |  |  |
| αSMA | Mouse, monoclonal | Novus | NBP2-33006 | 1/400 |
| PDGFRα | Rabbit, monoclonal | Cell Signaling Technology | #3174 | 1/200 |
| *Secondary antibodies* as above |  | Thermo Fisher Scientific |  | 1/800 |
