# Supplementary material for: Fibroblast-induced mammary epithelial branching depends on fibroblast contractility
Source: PLoS Biol. 2024 Jan 10;22(1):e3002093. doi: 10.1371/journal.pbio.3002093 (PMC10805323; doi:10.1371/journal.pbio.3002093)
Supplement: S3 Table — (DOCX) [file pbio.3002093.s022.docx]

**Supplementary Table 3. The list of primers used for qPCR in this study.**

| **Gene name** | **Forward primer**  **(5'-3')** | **Reverse primer**  **(5'-3')** | **length [bp]** |
| --- | --- | --- | --- |
| ***Actb*** | GGCTGTATTCCCCTCCATCG | CCAGTTGGTAACAATGCCATGT | 154 |
| ***Eef1g*** | TTCCTGCCGGCAAGGTTCCA | TGCCGCCTCTGGCGTACTTC | 119 |
| ***Myh9*** | GGCCCTGCTAGATGAGGAGT | CTTGGGCTTCTGGAACTTGG | 106 |
| ***Myh10*** | GGAATCCTTTGGAAATGCGAAGA | GCCCCAACAATATAGCCAGTTAC | 102 |
| ***Myh14*** | CAGTGACCATGTCCGTGTCTG | CGTAGAGGAACGATTGGGCTG | 81 |
