# Supplementary material for: Fibroblast-induced mammary epithelial branching depends on fibroblast contractility
Source: PLoS Biol. 2024 Jan 10;22(1):e3002093. doi: 10.1371/journal.pbio.3002093 (PMC10805323; doi:10.1371/journal.pbio.3002093)
Supplement: Attachment — Submitted filename: Response to Reviewers.pdf [file pbio.3002093.s023.pdf]

Manuscript ID PBIOLOGY-D-23-00673R1

Response to the Reviewers

Reviewers' comments:

Reviewer 1:

In this highly interesting and intriguing manuscript, Sumbal et al. describe how mammary fibroblast change the behavior of mammary epithelial organoids and induce budding/branching in the absence of commonly used "branching inducers" such as Fgf2. This induction was dependent on the physical contact between fibroblasts and mammary epithelial cells, as well as fibroblast contractility. Thereafter, the authors provide evidence that this fibroblast-dependent branching behavior is associated with patterned YAP and ERK activity.

This study is thought provoking and proposes an interesting novel concept. The finding that co-culture of contractile fibroblasts in breast cancer spheroids (that normally do not branch) reconstitutes budding morphogenesis nicely substantiates and expands the findings made with primary mammary organoids. Disappointingly, however, it remained unclear how well the proposed mechanism reflects branching morphogenesis in vivo. Also, certain aspects of the study lack quantitative analysis that are needed to support the conclusions.

We thank the Reviewer for finding our study interesting and for recognizing the novelty of the mechanism described in our study. As for the listed shortcomings of the manuscript, we provide new data that support the relevance of the described mechanism for the branching morphogenesis *in vivo* (shown below in response to the specific issues raised) and we performed the requested quantitative analyses.

Major issues:

My main concern is to what extent the observed phenomenon recapitulates in vivo situation, as this remains completely open. Some in vivo analysis would greatly strengthen the manuscript to support the main conclusion and to reduce the likelihood of an in vitro artefact. For example, how are fibroblasts located and organized in vivo in growing and bifurcating TEBs? Or in side branches? Can 'fibroblast loops' be observed in vivo?

We agree with the Reviewer that *in vivo* data on fibroblast localization around TEBs would greatly advance the manuscript by providing evidence for the relevance of the described mechanism for *in vivo* situation. Therefore, we have performed such investigation and now we can provide images from 3D imaging of whole TEBs and associated stroma from pubertal mouse mammary gland with immunostained fibroblasts (marker vimentin, VIM) as well as a set of histological mammary gland sections (Reviewer Figure R1, Figure 6 in the revised manuscript).

These data provide evidence that fibroblasts (immunodetected by their marker PDGFRα) accumulate mostly at the neck of TEBs in pubertal mammary glands. We uncovered that the fibroblasts around the TEB neck specifically express contractility marker α smooth muscle actin (αSMA), while the fibroblasts localized around fully formed ducts do not express αSMA (Reviewer Figure R1 A, B; Figure 6 A,B in the revised manuscript). Importantly, fibroblasts in co-cultures with organoids express αSMA, too (Reviewer Figure R1C; Figure 6C in the revised manuscript). Moreover, ***in vivo* the fibroblasts are oriented perpendicularly to the growth direction of mammary epithelium, forming loops in the bifurcation zone of the TEB and at the neck of the growing TEB** (Reviewer Figure R1 D, E; Figure 6D, E in the revised manuscript), **similarly to the loops observed in organoids** (Reviewer Figure R1E; Figure 6E in the revised manuscript). These new results are described in the revised manuscript in lines 255-268.

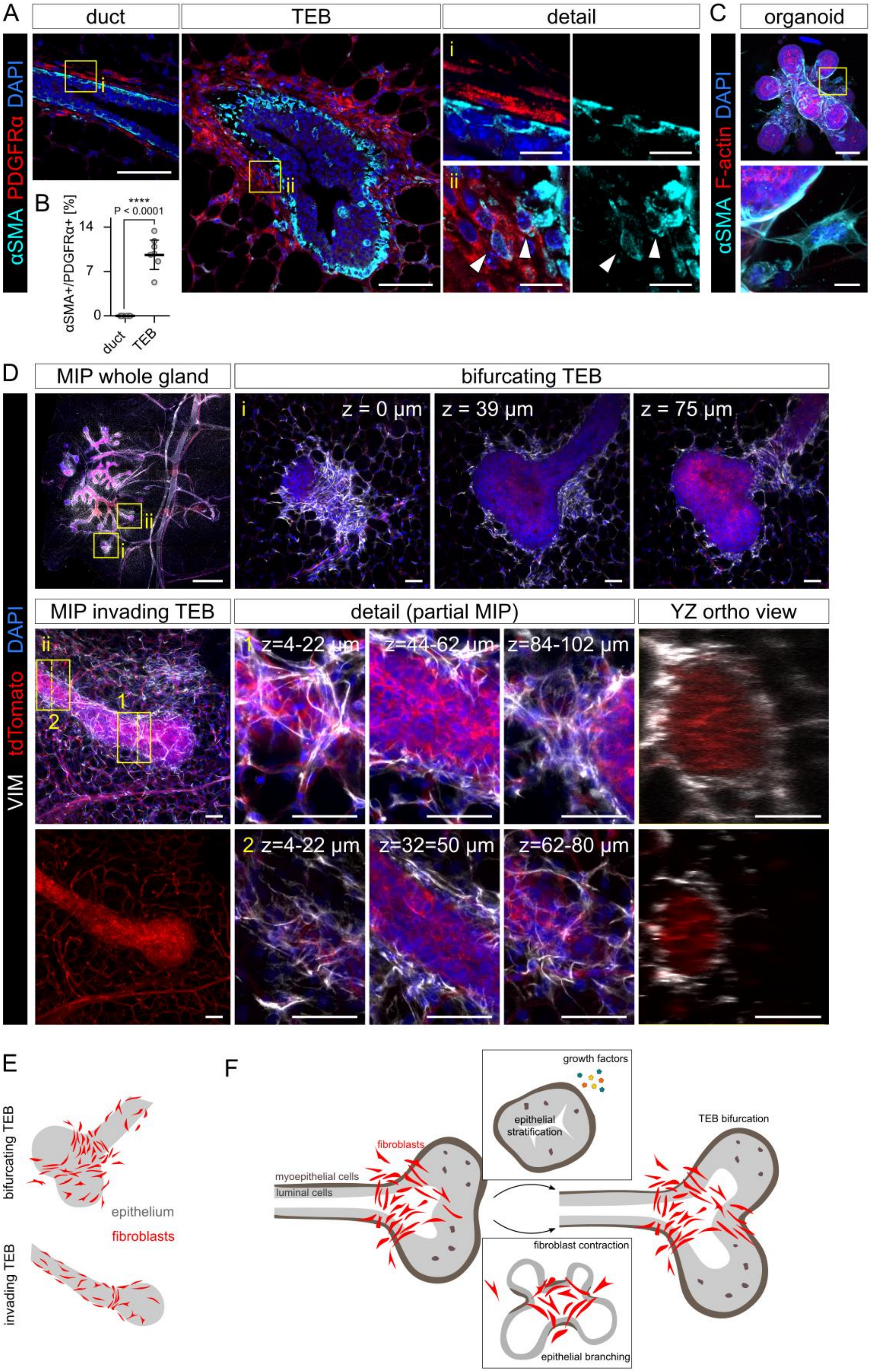
A
duct
TEB
detail
C
organoid
αSMA PDGFRα DAPI
i
ii
B
****
P < 0.0001
αSMA+/PDGFRα+ [%]
14
7
0
duct
TEB
αSMA F-actin DAPI
D
MIP whole gland
bifurcating TEB
z = 0 µm
z = 39 µm
z = 75 µm
MIP invading TEB
detail (partial MIP)
YZ ortho view
VIM tdTomato DAPI
1
2
z=4-22 µm
z=44-62 µm
z=84-102 µm
z=32=50 µm
z=62-80 µm
E
bifurcating TEB
invading TEB
epithelium
fibroblasts
F
growth factors
epithelial stratification
fibroblasts
myoepithelial cells
luminal cells
TEB bifurcation
fibroblast contraction
epithelial branching

Reviewer Figure R1: Contractile fibroblasts surround mammary TEBs in vivo. A. PDGFRα and αSMA staining on pubertal mammary gland sections and detail of peri-ductal and peri-TEB fibroblasts. Scale bar: 50 μm and 10 μm in detail. **B.** Quantification of αSMA+ cells out of PDGFRα+ fibroblasts. The plot shows mean ± SD, each dot represents one field of view, n = 7 TEBs and 8 ducts; statistical analysis: *t*-test. **C.** αSMA staining in a dispersed co-culture (day 5) and a detail of an αSMA+ fibroblast in contact with the organoid. Scale bar: 50 μm; scale bar in detail 10 μm. **D.** Whole-mount imaging of pubertal mammary gland stained for vimentin (VIM, a fibroblast marker). Detail (i) shows a bifurcating TEB. Detail (ii) shows an invading TEB, with close-up showing partial maximum intensity projections (MIPs) of upper, middle and lower part of the TEB neck (1) and a subtending duct (2). The positions of the orthogonal YZ views are indicated with dashed yellow lines. Scale bar: 1 mm in the whole gland MIP, 50 μm in other images. **E.** A schematic representation of fibroblasts surrounding TEBs *in vivo* and an organoid *in vitro.* Schemes were drawn from Figure 6D and Figure 3B. **F.** A schematic representation of our hypothesis on the role of contractile fibroblasts in TEB branching. Two mechanisms, paracrine signaling (growth factors) and mechanical cues (fibroblast contractility), which we uncoupled *in vitro*, work together to support mammary branching morphogenesis (TEB bifurcation) *in vivo*.

The authors make the point that the organoids cultured with fibroblasts retain a bilayer structure (though without full basal cell coverage, if I understood it correctly (Figure 3C), hence not recapitulating the in vivo epithelium in this respect). However, TEBs are multicellular stratified structures, yet the authors discuss that their model is a model for TEB bifurcation. To me this is counter-intuitive given the very different epithelial structure of TEBs and the organoids described in this study. What evidence can the authors provide to support their conclusion that their system models TEB clefting?

The Reviewer correctly points out that *in vivo* TEBs are composed of a stratified epithelium, reaching 5-10 layers of luminal (body) cells, and that such stratification is not always observed in our organoid-fibroblasts co-cultures. However, there is currently no mammary organoid model able to fully recapitulate the TEB phenotype. Classic mammary organoids cultured in Matrigel with FGF2 mimic epithelial stratification to some extent (reaching 3-4 layers of "body" cells in "TEB-like" ends of the branches) but do not support full myoepithelial coverage of branches (Nguyen-Ngoc and Ewald, 2013). Addition of fibrillar collagen to the cultures promotes full myoepithelial coverage of branches and their elongation (Nguyen-Ngoc and Ewald, 2013) but diminishes stratification. Alternating supplementation with FGF2 and EGF promotes further elongation and secondary branching (Caruso et al., 2022) but again, epithelial stratification remains limited to a few layers.

In vivo the TEBs are surrounded by a complex stroma, which provides instructions for epithelial morphogenesis, including several stromal cell types besides fibroblasts (adipocytes, immune cells) that secrete paracrine signals for epithelial proliferation (Gouon-Evans et al., 2000; Gouon-Evans et al., 2002; Gyorki et al., 2009; Lilla and Werb, 2010; Parsa et al., 2008; Sferruzzi-Perri et al., 2003). However, in the co-cultures used in this study we model only a part of the complex *in vivo* setting, specifically the role of contractile fibroblasts, which likely interact with the other stromal signals that are missing in our reductionist *in vitro* model. The **use of a minimal basal medium in co-cultures enabled us to untangle the importance of the cell contact from paracrine signaling**, which is the major original point of our study.

To test our hypothesis that *in vivo* it is the combination of both paracrine signaling (that supports epithelial stratification) and of fibroblast contractility (that guides epithelial branching), which together regulate TEB bifurcation, **we performed an experiment where we treated the co-cultures with a stabilized form of FGF2 [FGF2-STAB;** (Dvorak et al., 2018; Koledova et al., 2019)**]** that we had previously reported as **a strong proliferative factor for mammary organoids, triggering a TEB-like morphogenesis** of mammary organoids (Sumbal et al., 2020). The combination of fibroblasts and FGF2-STAB led to bigger organoid branches composed of stratified luminal cells, fully covered by myoepithelial cells (KRT5+) and containing sporadic

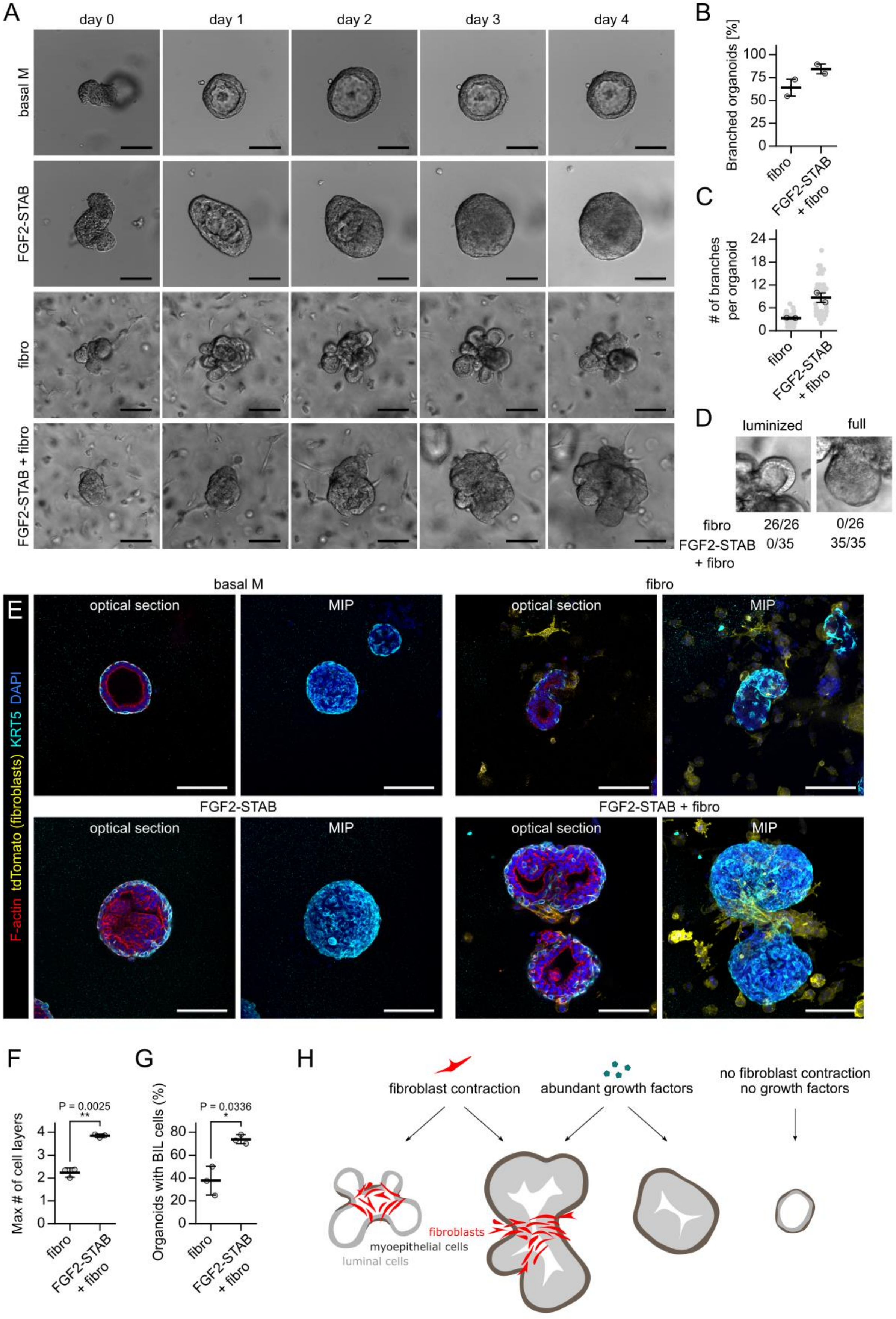

A
day 0
day 1
day 2
day 3
day 4
basal M
FGF2-STAB
fibro
FGF2-STAB + fibro
B
Branched organoids [%]
100
75
50
25
0
fibro
FGF2-STAB + fibro
C
# of branches per organoid
24
18
12
6
0
fibro
FGF2-STAB + fibro
D
luminized
full
fibro 26/26 0/26
FGF2-STAB + fibro 0/35 35/35
E
basal M
fibro
optical section
MIP
FGF2-STAB
FGF2-STAB + fibro
F-actin tdTomato (fibroblasts) KRT5 DAPI
F
Max # of cell layers
P = 0.0025
**
4
3
2
1
0
fibro
FGF2-STAB + fibro
G
Organoids with BIL cells (%)
P = 0.0336
*
80
60
40
20
0
fibro
FGF2-STAB + fibro
H
fibroblast contraction
abundant growth factors
no fibroblast contraction
no growth factors
fibroblasts
myoepithelial cells
luminal cells

Reviewer Figure R2. Combination of fibroblasts and FGF2-STAB induces TEB-like phenotype of organoids. A. Time-lapse snap shots of organoids grown in basal organoid medium with no exogenous growth factors (basal M), with FGF2-STAB, co-cultured with fibroblasts or co-cultured with fibroblasts with FGF2-STAB. Scale bar: 100 µm. **B.** Quantification of organoid branching. The plot shows mean ± SD. n = 2 independent biological replicates, N = 20 organoids per experiment. **C.** Quantification of number of branches per branched organoid. The plot shows mean ± SD. n = 2 independent biological replicates, N = 12-19 branching organoids per experiment. **D.** Examples of a luminized and a full branch on bright-field imaging and quantification of the branch phenotypes. n = 2 independent biological replicates, N = 12-19 branching organoids per experiment. **E.** Representative confocal images of organoids on day 5 of culture with FGF2-STAB or fibroblasts (dispersed co-culture). Scale bar: 100 µm. **F.** Quantification of maximum number of cell layers in a branch in confocal images. The plot shows mean ± SD. The dots represent averages from individual experiments. Statistical analysis: two-tailored *t*-test; n = 3 independent biological replicates, N = 9-13 organoids per experiment. **G.** Quantification of the percentage of organoids with KRT5+ cells present within the layers of KRT5- cells (basal-in-luminal, BIL cells) in confocal images. The plot shows mean ± SD. Statistical analysis: two-tailored *t*-test; n = 3 independent biological replicates, N = 9-13 organoids per experiment. **H.** A schematic representation of uncoupling fibroblast contraction and growth factor signaling in organoids.

KRT5+ cell in between luminal cell layers [similarly to cap-in-body cells in TEBs (Paine and Lewis, 2017) (Reviewer Figure R2; Figure 5 in the revised manuscript).These results demonstrate that **the combination of contractile forces exerted by fibroblasts and proliferative signals from the stroma can recapitulate TEB-like structures in organoid cultures.** These new results are described in the revised manuscript in lines 243-254.

Specific comments:

1. Lines 138-139 state: "On the branched organoids, fibroblasts were exclusively located around the necks of the nascent branches … (Figure 3B)."

Lines 255-256: "Our work reveals that mechanical strain imposed on mammary epithelial cells by fibroblasts results in epithelial folding with negative curvature in the epithelial-fibroblast contact points."

These are strong statements, however, these conclusions are not evident to me based on the images and should be backed-up with quantifications on fibroblast locations with respect to the co-cultured organoids. In fact, Movie 1 rather seems to suggest that fibroblasts arrive once the negative epithelial curvature is already emerging. Can the authors exclude the possibility that fibroblasts stabilize, rather than induce the formation of new buds? Quantification of the fibroblast locations in fixed samples and/or quantifications of their behaviors in the time-lapse movies would be needed to support these conclusions that are critical to the manuscript.

We thank the Reviewer for pointing this out and we agree that in the bright-field movies, it may be difficult to judge the exact fibroblast localization once the fibroblasts "sit" on the organoid. However, in Movie 1, it can be observed that several fibroblasts make contact with the organoid between 0-50 h of the experiment, when the overall shape of the organoid is still round, with no negative curvature present. It is right at these contact points with fibroblasts where the epithelium will start bending to negative curvatures and forming branches. Importantly, in the absence of fibroblasts (or a potent branching-inducing factor, such as FGF2), epithelial organoids never branch, neither the epithelium bends to negative curvatures.

To clarify this issue, **we now present a day-by-day quantification of fibroblast contact points with the epithelium extracted from the time-lapse movies in relation to the organoid's circularity and newly formed branches** (Reviewer Figure R3 A-C; Suppl. Figure 2

A-C in the revised manuscript), **as well as quantification of fibroblast-organoid contact points in fixed co-cultures with labeled fibroblasts** (Reviewer Figure R3 D, E; Suppl. Figure 2 D, E in the revised manuscript). These new results are included in the revised manuscript in lines 143-145 and 148-152, respectively.

Also, we provide quantification of the imaging in Figure 3B in the paper (Reviewer Figure R4), as a revised part of Figure 3. We accordingly adjusted our conclusions in the manuscript to "On the branched organoids, fibroblasts were predominantly located around the necks of the nascent branches" (lines 153-154).

Importantly, the fibroblasts play a role also in stabilization of the branches they induced – see response to the point 2 of Reviewer 1 below.

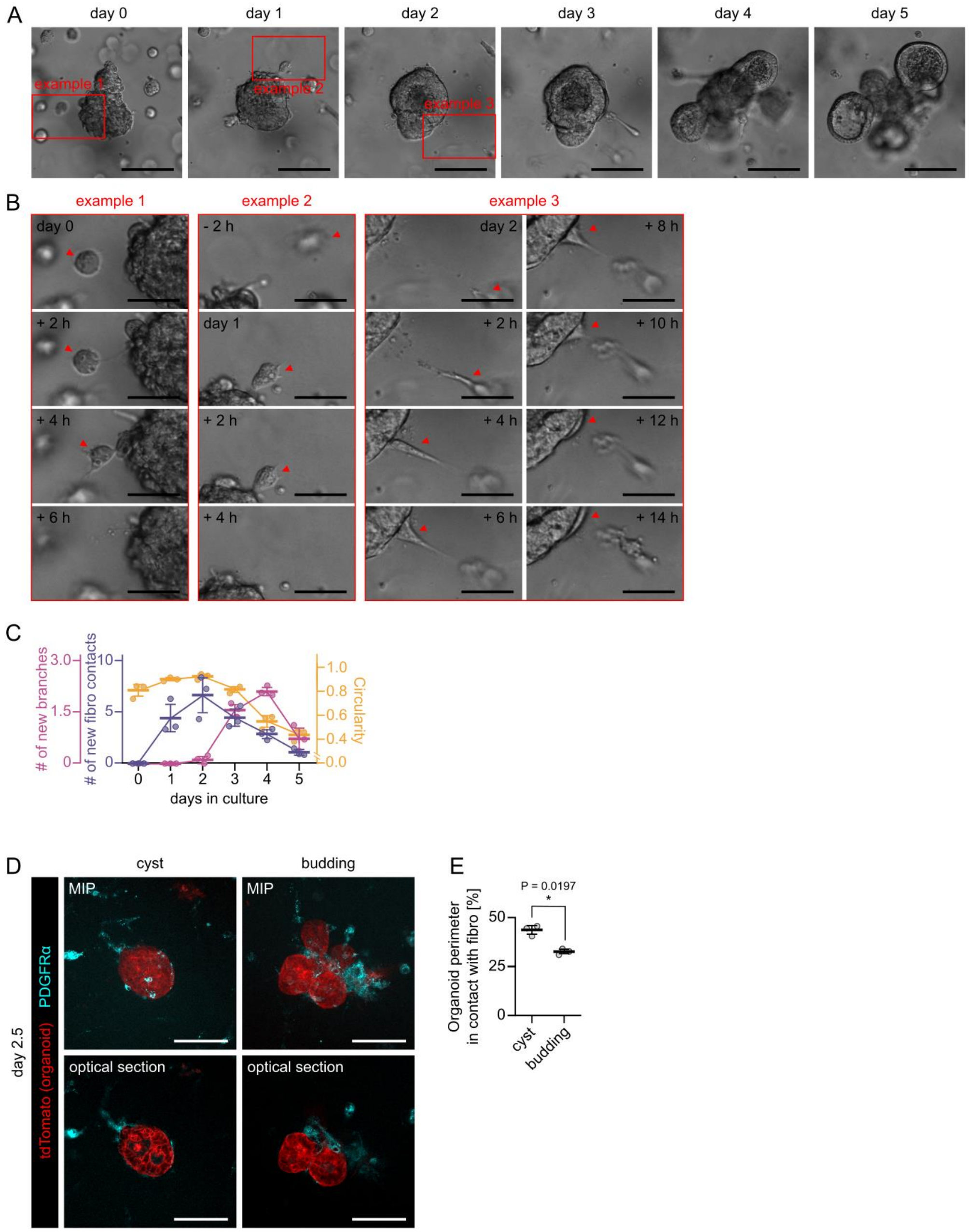

Reviewer Figure R3: Fibroblast-organoid contacts precede organoid branching. A. Time-lapse snapshots of an organoid-fibroblast dispersed co-culture. Scale bar: 100 µm. **B.** Detailed snapshots of three

examples of fibroblast-organoid contact establishment in the co-cultures shown in **A** on day 1, 2 and 3. Red arrowheads indicate fibroblasts of interest. Scale bar: 50 µm. **C.** Quantification of organoid circularity (data from **Figure 1**), number of new branches and number of established fibroblast-organoid contacts from matched experiments. The plot shows mean ± SD; n = 3 (each dot represents the average from a biologically independent experiment, N= 20 organoids per experiment). **D.** Maximum intensity projection (MIP) and optical section images of a dispersed co-culture on day 2.5, representative images of cystic and budding organoids (tdTomato). Fibroblasts were detected by immunostaining for PDGFRα. Scale bar: 100 µm. **E.** Quantification of organoid middle section perimeter in contact with PDGFRα signal. The plot shows mean ± SD. Each dot represents an average from one experiment. Statistical analysis: two-tailored *t*-test; n = 3 independent biological samples, N = 15-24 organoids per sample.

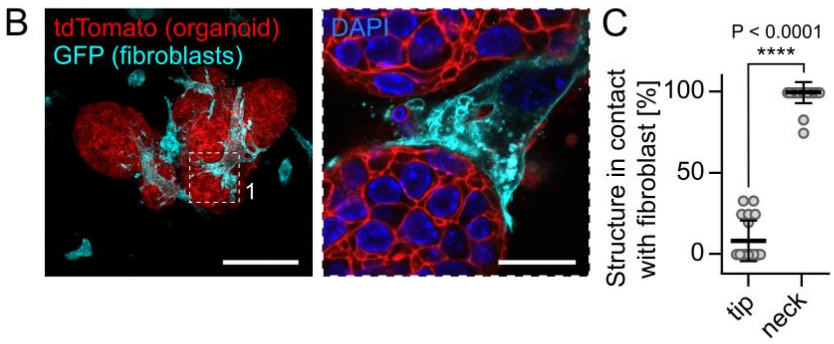

Reviewer Figure R4: Fibroblasts in co-cultures are in physical contact with the epithelium. B, C. Images (**B**) and quantification (**C**) of the contact point between organoid (tdTomato) and fibroblasts (GFP) on day 4 of co-culture (dispersed culture). Scale bar: 100 µm, scale bar in detail: 20 µm. **C.** The plot shows mean ± SD, each dot represents one organoid, n = 5 experiments, N = 21 organoids. Statistical analysis: Two-tailored *t*-test.

---

2. Functional data on the importance of fibroblast contractility (Fig. 4C-E) is convincing. Yet, contractility could also be related to cell motility. I see many of the fibroblasts "swimming" toward the epithelium in the videos provided, but what about the Myh9 ablated ones? Can the authors exclude the possibility that they fail to move toward the organoid? A movie similar to Movie 2 but using Myh9 deleted fibroblasts would be informative (fibroblasts visualized e.g. using live dyes). An alternative option would be to perform live imaging of organoids that are already undergoing branching followed by blebbistatin/ROCK inhibitor treatment and further imaging. How does the perturbation change fibroblast behaviors?

We agree with the Reviewer that contractility is often linked to cell motility and we understand the concern. However, we observed that upon inhibition of contractility with the ROCK inhibitor Y-27632 or with blebbistatin, the fibroblasts still actively migrated in Matrigel [in fact, Y-27632 as well as blebbistatin increase fibroblast migration in Matrigel, as we previously reported (Sumbal and Koledova, 2019)] and were able to contact the organoid. Moreover, in Movies 4 and 5, it is evident that *Myh9* ablated fibroblasts are motile and able to reach the organoid. **We provide the quantification** of fibroblast-organoid contact establishment (as a result of fibroblast migration toward the organoids) upon Y27632/blebbistatin treatment (Reviewer Figure R5; Suppl. Figure 5 in the revised manuscript) or adenovirus-mediated *Myh9* knockdown (Reviewer Figure R6; Suppl. Figure 9 in the revised manuscript). In sum, **contractility inhibitors or *Myh9* knockout did not diminish the capability of fibroblasts to migrate towards and contact the organoids**. These new results are described in the revised manuscript in lines 175-178 and 187-194, respectively.

To address the alternative suggestion "to perform live imaging of organoids that are already undergoing branching followed by blebbistatin/ROCK inhibitor treatment and further imaging. How does the perturbation change fibroblast behaviors?". We believe that such an

experiment would not help to address the Reviewer's concern regarding fibroblast migration because most movement towards the organoids happens within the first 48 h of co-culture, when the branches are not yet visible (see also quantification in Reviewer Figure R3C). Adding contractility inhibitors when the organoids are already branching thus would not show an effect on fibroblast migration. However, **we performed the proposed experiment** (blebbistatin or ROCK inhibitor were added on day 3 of the co-culture, when branches were already being formed) to test if fibroblasts play a role in branch stabilization (or maintenance). We observed branch loss/retraction in 20-40% organoids, suggesting that **fibroblast contractility is necessary also for branch stabilization**. We now provide these results in Reviewer Figure R7 (Suppl. Figure 6 in the revised manuscript) and in the Result section of the revised manuscript, lines 178-181.

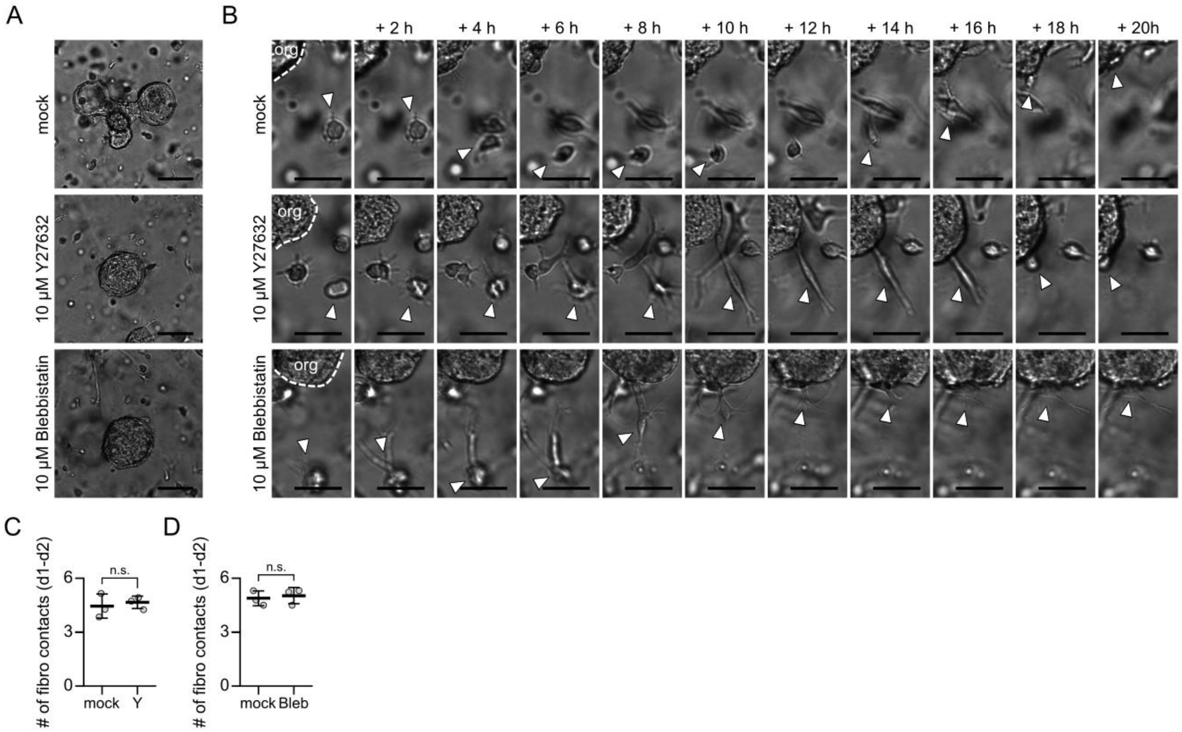

Reviewer Figure R5: Contractility inhibitors do not impede fibroblast motility. A. Representative endpoint images of organoid in co-cultures with contractility inhibitors. Scale bar: 100 µm. **B.** Detailed time-lapse snapshots of fibroblast-organoid contact establishment in co-cultures with the inhibitors. Scale bar: 50 µm. White arrowhead indicates the fibroblast of interest. **C, D.** Quantification of fibroblast-organoid contacts established in co-cultures with inhibitors (Y = 10 µM Y27632 (**C**); Bleb = 10 µM Blebbistatin (**D**)) within the first 2 days. The plots show mean ± SD. Statistical analysis: two-tailored t-test; n = 3 independent biological replicates, N = 10 organoids per experiment.

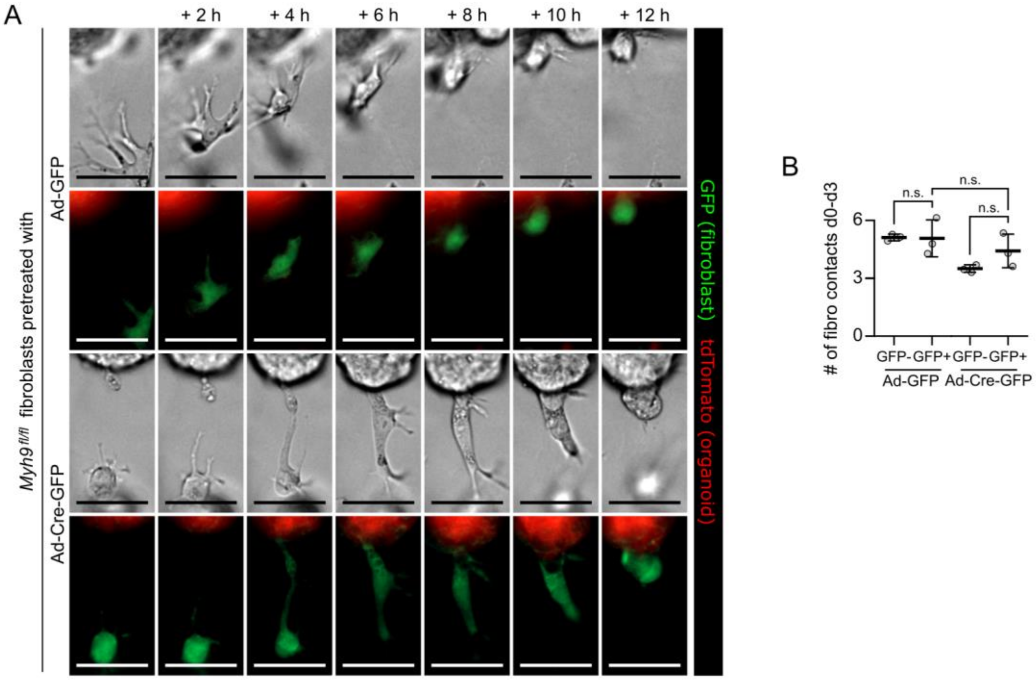

Reviewer Figure R6: *Myh9* knock-out does not impede fibroblast motility. A. Detailed time-lapse snapshots of fibroblast-organoid contact establishment in dispersed co-cultures with control or *Myh9*-KO fibroblasts and tdTomato+ organoids. Scale bar: 50 µm **B.** Quantification of fibroblast-organoid contacts established in the first 3 days of co-culture, comparing GFP+ and GFP- fibroblasts (GFP is a marker of adenoviral transduction). The plot shows mean ± SD. Statistical analysis: two-tailored *t*-test; n = 3 independent biological replicates, N = 20 organoids per experiment.

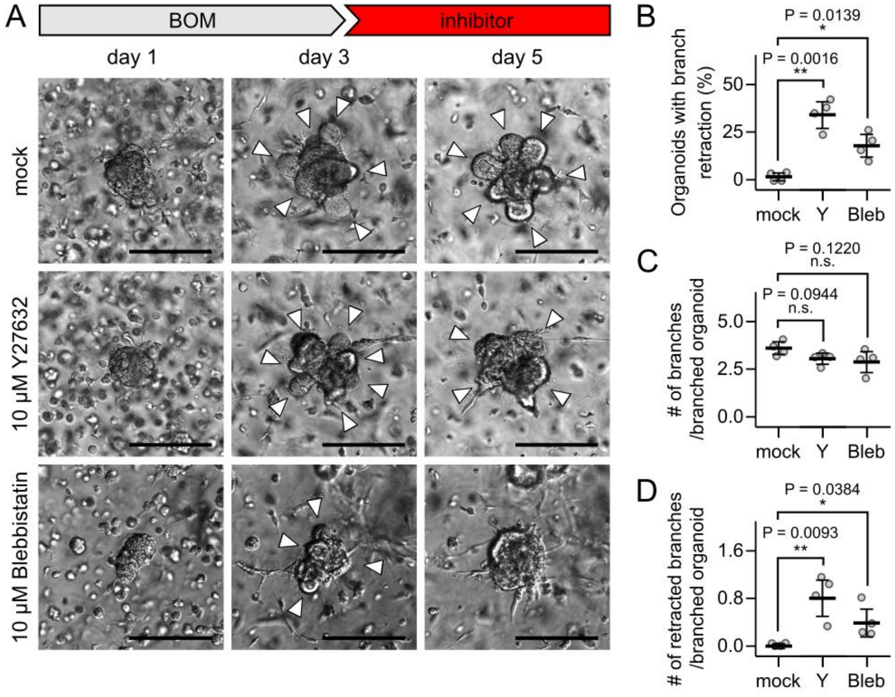

Reviewer Figure R7: Fibroblast contractility is required for branch stabilization. A. Experimental scheme and time-lapse snapshots of co-cultures treated with contractility inhibitors on day 3 of culture. Scale bar: 100 µm. White arrowheads indicate organoid branches. **B-D.** Quantification of organoids with retracted branches (**B**), number of formed branches per branched organoids (**C**) and number of retracted branches per organoid (**D**). The plots show mean ± SD. Statistical analysis: two-tailored *t*-test; n = 4 independent biological replicates, N = 20 organoids per experiment.

The authors also describe "cellular loops" generated by fibroblasts (Figure 4A). However, Fig. 4A looks somewhat different from Fig. 3B that also shows the location of fibroblasts. Could the authors provide quantifications on how often such 'cellular loops' were detected?

We thank the Reviewer for this observation. The difference is probably due to a less complex shape of the organoid in Figure 4A than of the organoid in Figure 3B, therefore, the loops are more clearly visible. **We provide quantification on the detection of cellular loops** in Reviewer Figure R8 (Suppl. Figure 3B, C in the revised manuscript) and describe the results in lines 164-166 of the revised manuscript.

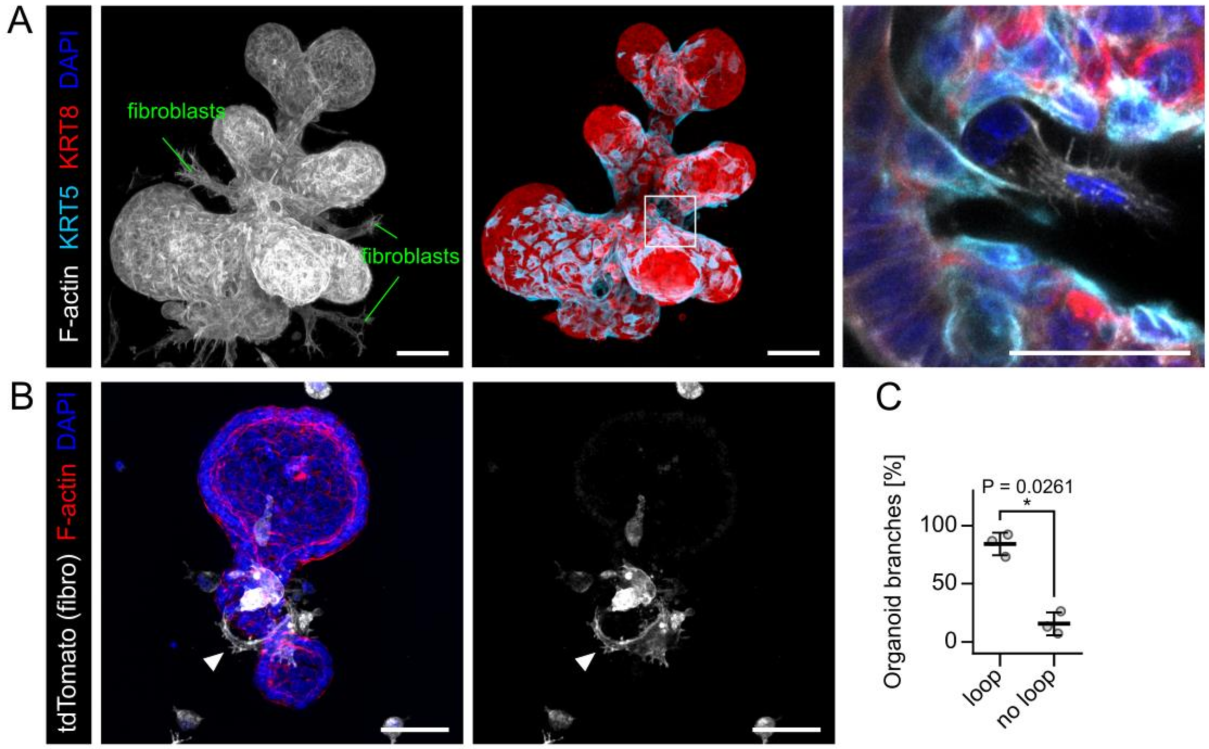

Reviewer Figure R8: Quantification of fibroblast loops. A. A representative confocal image of a dispersed co-culture on day 4. Scale bar: 20 µm, scale var in detail: 10 µm. **B.** A representative confocal image of dispersed organoid-fibroblast co-culture on day 3. The line indicates the fibroblast loop at the branch neck. Scale bar: 50 µm. **C.** Quantification of organoid branches in co-cultures with or without the fibroblast loop present. The plot shows mean ± SD. Statistical analysis: two-tailored *t*-test; n = 3 independent biological replicates, N = 5-12 organoids per experiment. 56 branches in total.

3. The authors make the point that "fibroblast-induced branching requires epithelial proliferation". I am not fully convinced by the importance of these findings (Fig. 5E-F) - doesn't Fgf2 -dependent branching also require proliferation? Aren't new cells needed to build new branches?

We fully agree with the Reviewer that epithelial proliferation is also necessary for FGF2-dependent branching; however, Huebner et al. showed that proliferation is NOT necessary for branch elongation (Huebner et al., 2016). We do not claim that the requirement of epithelial proliferation represents the critical difference between FGF2- and fibroblast-induced branching mechanisms. The purpose of the results shown in Fig. 5E-F was to demonstrate that epithelial organoids grown in co-culture are not simply passively "being squeezed" and reshaped by contractile fibroblasts, but instead they play an active role in building new branches.

The authors also make the point of patterned cell proliferation and show that the stalk is more proliferative compared to the tip. Do the authors consider this a physiologically relevant finding? In other words, does this fit with the data from TEBs?

TEBs *in vivo* are the structures where the vast majority of epithelial proliferation is confined (Scheele et al., 2017), and they are more proliferative than organoids in co-cultures. As discussed in response to the first point of Reviewer 1, this difference is probably due to the *in vitro* lack of additional paracrine growth factors, which are provided by other stromal cells. However, it was demonstrated that although the whole TEB contains proliferative cells, it is the cells localized in the "stalk"/neck of the TEB (which we found surrounded by contractile fibroblasts; Reviewer Figure R1), which will mainly contribute to the growth of the adjacent duct, and not the cells at the TEB tip (Scheele et al., 2017). We now discuss this in the revised manuscript in lines 376-380.

If patterned proliferation is linked with presence of fibroblasts, then presumably it is not observed in Fgf2-induced branches? This should be assessed.

In the revised manuscript, **we provide imaging and quantification of EdU+ cells in FGF2-induced branches.** As correctly expected by the reviewer, we did not observe the same pattern of proliferation in FGF2-induced branches (Reviewer Figure R9; Suppl. Figure 10 A-D in the revised manuscript).

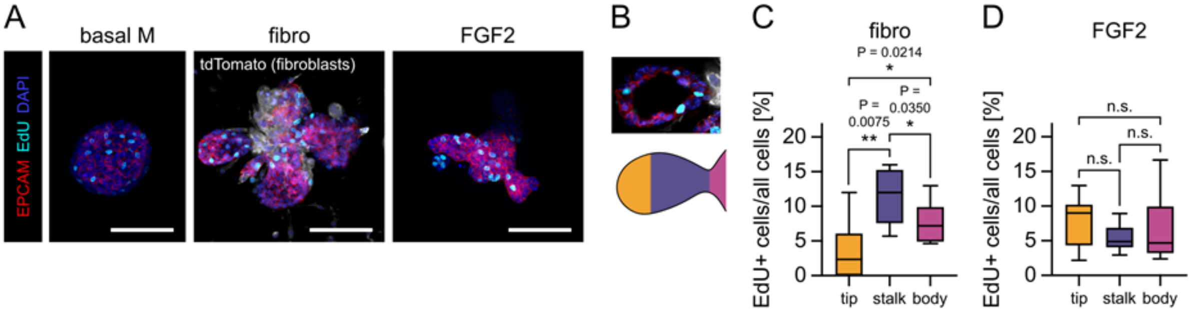

Reviewer Figure R9: Proliferation in co-culture system. A. representative images of organoids on day 4 of culture in basal medium (basal M), in dispersed co-culture with fibroblasts, or with FGF2. EdU was administered 2 h pre-fixation; EPCAM (red), DAPI (blue), EdU (cyan), fibroblasts were isolated from *R26-mT/mG* mice (tdTomato, white). Scale bar: 100 µm. **B.** Optical section of a branch from **A** (top), a scheme of branch regions (bottom). **C, D.** Quantification of percentage of EdU+ cells from epithelial cells in different branch regions in fibroblast-organoid dispersed co-culture (**C**) and in FGF2-treated organoid (**D**). The box and whiskers plot shows minimum, median, and maximum values, and second and third quartiles of data distribution. n = 3 independent experiments, N = 6 organoids, 2202 analyzed cells in **C**; N = 11 organoids, 3104 analyzed cells in **D**. Statistical analysis: Multiple *t*-tests.

4. The authors also show that YAP and ERK activity are patterned and based on the absence of patterning in co-cultures with Myh9-deleted fibroblasts, it is concluded that this depends on fibroblast contractility. However, under these conditions, branching does not take place, so this experimental approach is perhaps not the most informative one. What about Fgf2-induced branching: are YAP and ERK activities patterned differently compared to fibroblast co-cultured organoids?

We agree with the Reviewer's point and **performed P-ERK and YAP staining on FGF2-induced branching organoids** to distinguish whether the localization of signaling molecules is directly linked to the presence of fibroblasts, or whether it depends on the shape of the organoid as it is the case for intestinal organoids (Gjorevski et al., 2022). Our data show that the YAP signaling pattern that we observed in co-cultures is not present in FGF2-treated organoids (instead, cells with high/low YAP n/c ratio are randomly distributed), suggesting that increased YAP signaling at the branch neck is specific for the presence of fibroblasts (Reviewer Figure R10; Figure 4 G-I in the revised manuscript). These results are described in the revised manuscript, lines 215-216.

The P-ERK staining on FGF2-treated organoids shows a pattern with P-ERK high cells in clusters at the tips of branches (Reviewer Figure R11), in agreement with previous literature (Huebner et al., 2016). However, taking in consideration the comments of Reviewer 2 on ERK staining and dynamics, we decided to exclude the data on P-ERK staining from the manuscript, because the dynamic nature of ERK signaling is hard to explain by still images in co-cultures.

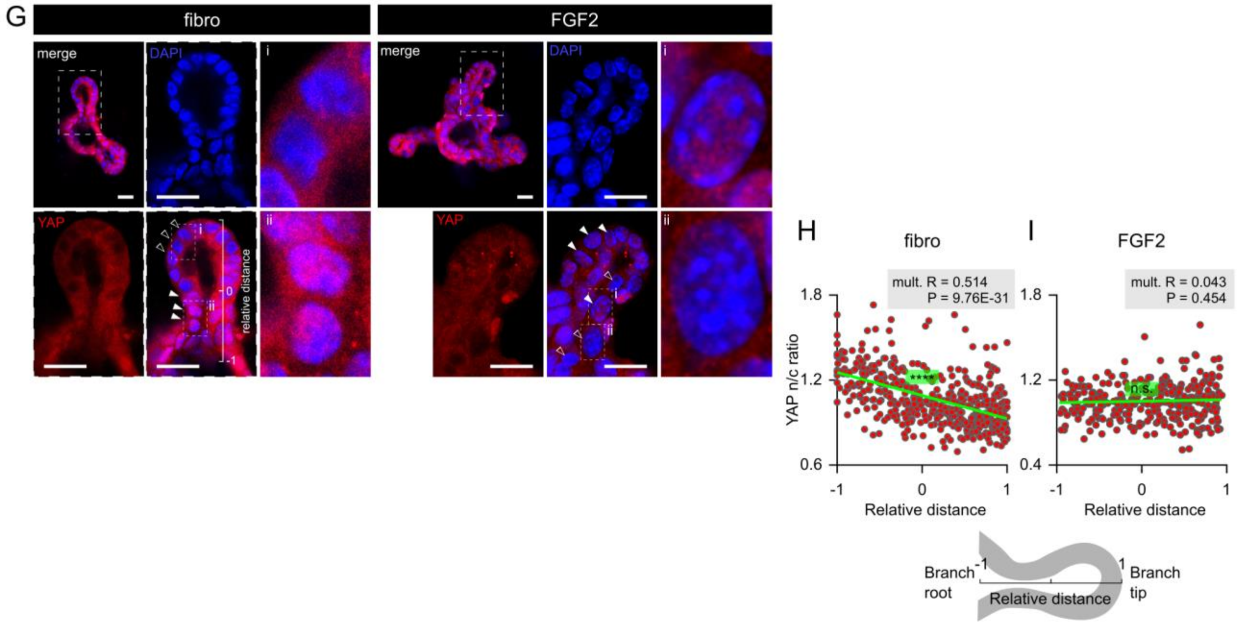

Reviewer Figure R10 (part of Figure 4 of the revised manuscript): G. Staining of YAP in an organoid co-cultured with fibroblasts (dispersed culture) or with FGF2. Scale bar: 20 µm. Ful arrowheads point to cells with nuclearly localized YAP, empty arrowheads point to cells with cytoplasmic YAP. **H, I.** Quantification of YAP nuclear/cytoplasmic signal ratio. The scheme explains relative distance: -1 is branch root, +1 is branch tip. Each dot represents a single cell, n = 436 cells from 19 branches of 10 organoids (fibro, **H**); n = 306 cells from 12 branches of 10 organoids (FGF2, **I**). Statistical analysis: Linear regression, mult. R indicates correlation coefficient; P is the result of ANOVA F-test.

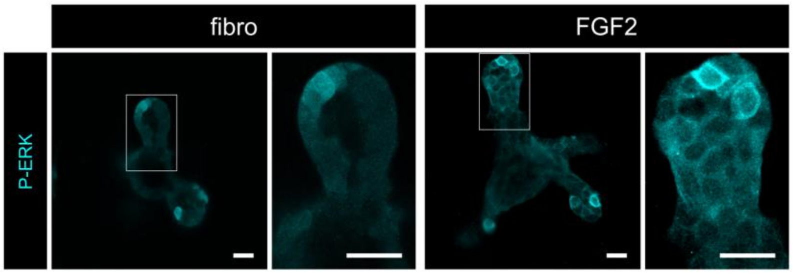

Reviewer Figure R11: P-ERK high cells accumulate at the branch tips. Staining of P-ERK in organoids co-cultured with fibroblasts or with FGF2. Scale bar: 20 µm.

---

5. The authors propose that the underlying epithelial cells can sense the contact with contractile fibroblasts as a mechanical stress leading to specific nuclear accumulation of YAP in the neck region of the buds. I am not sure if I am misinterpreting Figure 5I (a higher resolution close-up would be informative), but to me it looks there is a lot of nuclear YAP in organoids cultured without fibroblasts and Fgf2, which would argue that there is no such relationship between contractile fibroblasts and epithelial YAP. Instead, nuclear YAP might simply reveal and reflect the proliferative status of epithelial cells.

We agree with the Reviewer that nuclear YAP might reflect the proliferative status of cells because the Hippo-YAP/TAZ pathway is a regulator of cell proliferation (Dong et al., 2007; Huang et al., 2005; Ota and Sasaki, 2008). However, we would like to point out that in the discussed Figure 5I, the organoids in the lower panel, which contain several cells presenting nuclear YAP distributed all over the epithelium, are organoids co-cultured with *Myh9*KO fibroblasts. Under these conditions, the fibroblasts are providing paracrine signals for epithelial proliferation but lack contractile force for epithelial patterning. We present new data from organoids cultured without fibroblasts and without FGF2, which show that such organoids have minimum nuclear YAP and that nuclear YAP is only found in contractile basal cells (Reviewer Figure R12).

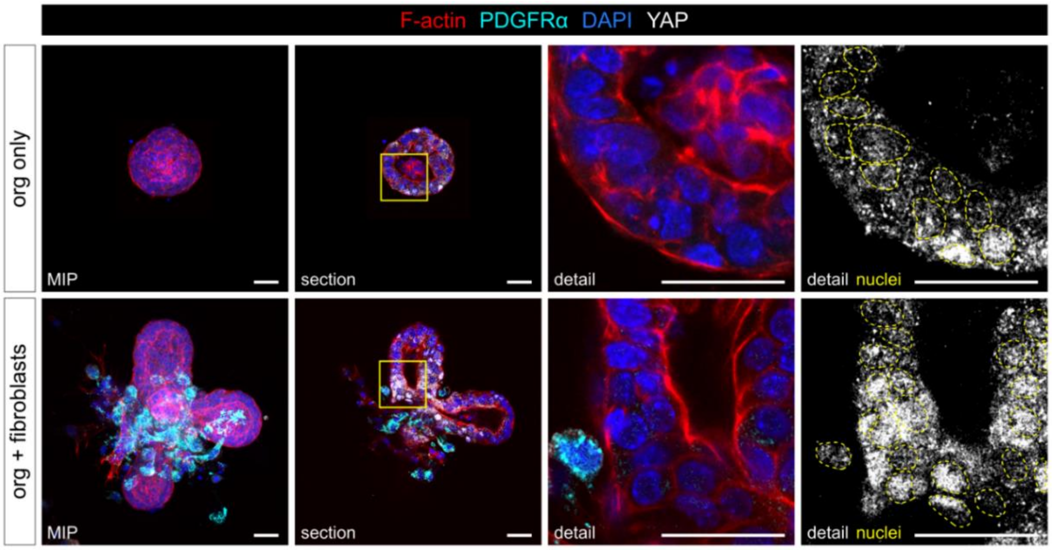

Reviewer Figure R12: YAP signaling activation in organoid-fibroblasts co-cultures. Maximum intensity projection, single optical section and a detail of an organoid cultured alone in basal medium or co-cultured with fibroblasts. In detail, note that in the organoid cultured alone only the basal cells (outer epithelial cells) present nuclear YAP, while in the organoid co-cultured with fibroblasts many cells have

nuclear YAP in the neck of the branch, where organoid is in contact with fibroblasts (PDGFRα). Scale bar: 50 µm.

Minor comments:

1. Line 86: "Organoids co-cultured with fibroblasts developed bigger but less numerous branches (Figure 1A, C)." It remained unclear to me where the size of the organoids was reported.

We apologize for the misunderstanding. With the cited statement, we meant that the branches were bigger but less numerous. We rephrased the sentence to make it clear: “First, organoids co-cultured with fibroblasts developed bigger branches, but the branches were less numerous” in the revised manuscript (lines 92-93) and **we provide quantification of branch size (thickness) of organoids in co-culture vs. with FGF2** (Reviewer Figure 13C; Figure 1C in the revised manuscript).

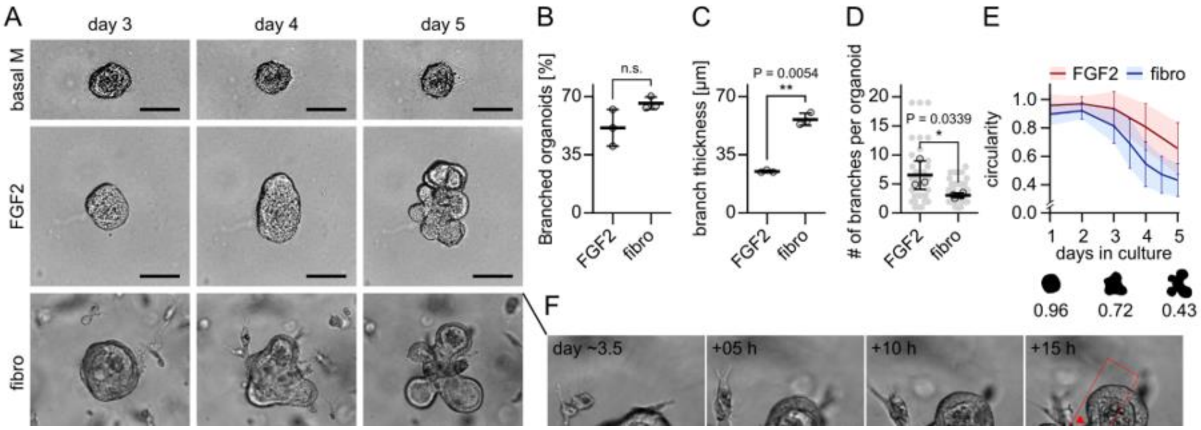

Reviewer Figure R13 (part of Figure 1 in the revised manuscript). C. Quantification of branch thickness from experiments in A. The plot shows mean ± SD, each dot represents a biologically independent experiment, n = 3. Statistical analysis: Two-tailored *t*-test.

2. The results and conditions used in Fig. 2 are very clear. However, thereafter it remained unclear whether the fibroblasts used in the experiments were preaggregated or not.

The pre-aggregation was only used in Figure 2, the rest of the experiments were performed in “standard” dispersed co-culture settings. We have specified this in the revised manuscript (mostly by adding “dispersed” throughout the Results and Figure legends, where applicable, to avoid confusion).

3. The correlation coefficient (linear regression) should be reported.

The correlation coefficient is reported together with ANOVA F-test for linear regression in the Figure 4 H, I.

4. Movie 2 could be more informative if it showed one branching event from the beginning to the end.

We agree. We repeated the experiment to show the whole branching event using tdTomato organoids and GFP-labeled fibroblasts. In the revised manuscript, we have updated the Figure 3 accordingly (Reviewer Figure 14; Figure 3A in the revised manuscript) together with new Movies that depict the whole branching event (Movie 2) and details of the fibroblast-organoid contact establishment (Movie 3).

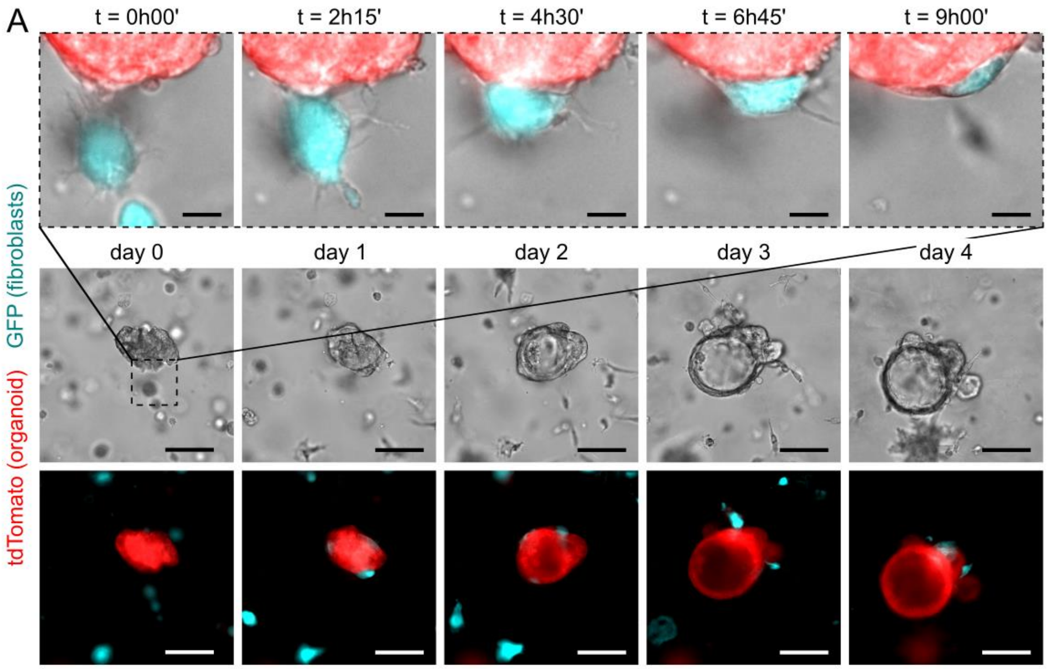

Reviewer Figure 14 for: Part of Figure 3 of the manuscript. A. Snapshots from time-lapse brightfield and fluorescence imaging of organoid (tdTomato) and fibroblast (GFP) co-culture (dispersed culture). Scale bar: 100 µm. Top line shows detail of fibroblast-organoid close interaction. Scale bar: 20 µm.

Reviewer 2:

This paper describes the morphogenesis of mammary organoids with respect to branching morphogenesis evoked by co-culture of fibroblast or incubation with the growth factor FGF2. They propose that fibroblasts shape branching morphogenesis by interacting with the organoids and providing an actomyosin-based constriction mechanism that participates in the shaping of the branches.

My first criticism is that the paper is not well written. As a reader with limited experience in mammary morphogenesis, I had the feeling that the authors expected me to know his organoid system as well as they do it. By example, the existence of myoepithelila cells in these organoid cultures (absent from simple epithelial cyst models such as MCF10A or MDCK) was never mentioned before discussed in the results (and this is clearly a strength of this model).

We appreciate the Reviewer's suggestion to make the paper more accessible to a wide audience by including a more detailed description of the strength of the organoid system, in particular the composition of both internal luminal and outer myoepithelial cells, reflecting the *in vivo* morphology of the mammary epithelium. We included this description in the Introduction in the revised manuscript (lines 69-70 in the revised manuscript).

The data in the paper are of good quality, but the different experiments are anecdotal and do not culminate in a paper with a clear message. Many perturbation experiments (such as the proliferation experiments in figure 5) cannot lead to the conclusions that the authors make. The experimental design is wrong from the beginning, leading to data that can only be mis- or over-interpreted. Finally, the discussion is hand waving, and the many results are not even discussed (the presumable interaction between the fibroblast and the myoepithelial cells). The finding that fibroblast contractility is required for fibroblast constriction around epithelia is trivial. I think it is the

striking interaction between the fibroblasts and the epithelium that is interesting, and should have been studied.

We apologize for the misunderstanding but we respectfully disagree with the Reviewer that many experiments do not lead to the conclusions we draw and that the findings that “fibroblast contractility is required for fibroblast constriction around epithelia is trivial”. In fact, **the major novelty of this paper lays in our original findings that the physical contact of fibroblasts with the epithelium and their contractility are necessary for mammary epithelial branching.** We would like to stress that such branching mechanisms are not trivial since they have not been described in the mammary gland before, and similar mechanisms of epithelial-stromal interactions have only recently started to be deciphered in other tissues.

We agree with the Reviewer that the interaction between the fibroblasts and the epithelium is interesting, and it should be further investigated in following-up studies.

We do not understand why the Reviewer finds that “many perturbation experiments (such as the proliferation experiments in figure 5) cannot lead to the conclusions that the authors make. The experimental design is wrong from the beginning, leading to data that can only be mis- or over-interpreted”. The perturbation experiments were carefully designed and employed inhibitors that are widely used by the scientific community (including the proliferation experiments in Figure 5). We discuss specific comments of the Reviewer to individual experiments in greater detail below.

We agree that the part on epithelial proliferation was not a major discovery (we never claimed it was). Accordingly, and to simplify the narrative, we have now moved the data on proliferation to supplementary information (Suppl. Figure 10 of the revised manuscript).

To me the paper is not of interest to the wide audience of PLOS biology. I would suggest to the authors to change the narrative of the paper to displaty the results (the images are of high quality) and send this to a specialized journal, or maybe PLOS one with major revisions.

We respectfully disagree with the Reviewer. We presented this unpublished work at broadly oriented developmental biology and mechanobiology international conferences and we were met with major interest from the wide scientific community.

Below some more specific comments.

Figure 1F is mentioned after 1G, cite figure panels in order of appearance
Figure 1G: denote what asterisks point to !

We thank the Reviewer for pointing out these issues. We have corrected them in the revised manuscript: we corrected labeling of Figure 1 panels and specified that the asterisks denote the lumen in the Figure 1 legend.

Line 127: Ras is a GTPase and not a kinase ! Usually one refers to the GTPase by mentioning its oncogenic mutation, as well as the Ras isoform: by example KRas G12V ?

We apologize for this oversight. We corrected “kinase” to “GTPase” and in the revised manuscript, we provide a reference for the cell line’s origin (Kasid et al., 1985) that indicates that the transfected oncogene was v-ras^{H}, which carries HRAS mutations G12R and A59T (Ruta et al., 1986).

Figure 3: the panels refer to mtomato and mGFP, the legend to tdtomato and gfp. What does mtomato or mGFP refer to ? Monomeric tomato ? Do the authors use a monomeric or a tandem tomato (I think monomeric tomtato does not even exist ?).

We thank the Reviewer for pointing this out and apologize for the confusion due to some inconsistencies in fluorophore nomenclature.”mtomato” and “tdtomato” indeed denote the same fluorophore carried in the *R26-mT/mG* mouse strain used to fluorescently label the cells (Muzumdar et al., 2007). “tdTomato” refers to “tandem tomato” because the fluorophore is a tandem dimer. “mTomato” refers to “membrane tomato” because the fluorophore is membrane-bound. Similarly, mGFP and GFP denote the same fluorophore, where “m” in the “mGFP” stands for “membrane” because it is membrane targeted. We corrected these inconsistencies in the nomenclature in the revised manuscript in both Figures and Legends).

Figure 3B: can the authors tell us what they see in panels 1,2,3. Are they just examples, or do the authors want to show us fibroblast - organoid interactions with specific features ! This reviewer feels like he has to guess what the authors want to tell him.

"On the branched organoids, fibroblasts were exclusively located around the necks of the nascent branches and sat directly in contact with the epithelium (Figure 3B). Is there a statistic about this ?

We apologize for not pointing out in the legend of original Figure 3B that the panels 1, 2, and 3 were examples of different contact points between the organoid and fibroblasts and showed various morphologies that the fibroblasts can adopt. We simplified the figure and now show only one example. Moreover, **we performed quantification of fibroblast distribution on the organoid** and provide it in this Response to Reviewers file as Figure 4 for the Reviewer above and in Figure 3C of the revised manuscript.

Figure 3C: interaction of fibroblasts with krt5 positive myoepithelial cells. It is difficult to judge if this interaction is just anecdotal, or a real phenomenon with n=1 example. Can the authors show us a statistic. The images are complex to understand.

The image provided in the original Figure 3C (Supplementary Figure 3A in the revised manuscript) is representative of multiple experiments (as all images in the manuscript). In the revised manuscript, we provide quantification of contact points between fibroblasts (stained with vimentin) and KRT5/KRT8 epithelial cells (Reviewer Figure R15).

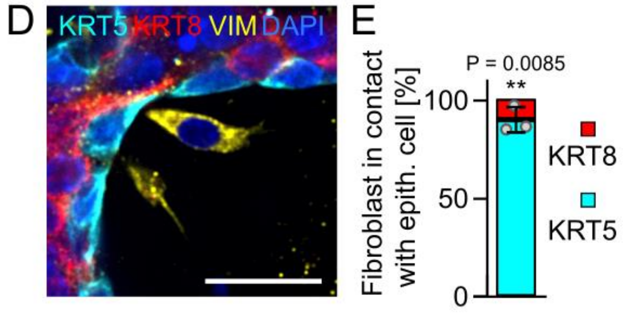

Reviewer Figure R15: Part of Figure 3 of the manuscript. D. Images of the contact point between organoid (luminal (KRT8) and myoepithelial (KRT5) cells) and fibroblasts (VIM) on day 5 of co-culture (dispersed culture). Scale bar: 20 µm. **E.** Quantification of fibroblasts in contact with KRT5+ or KRT8+ epithelial cells. The plot shows mean ± SD, each dot represents average from one biological replicate, n = 3 experiments, N = 14 organoids, 219 fibroblasts. Statistical analysis: Two-tailored t-test.

Also, not being familiar with the organoid culture system, I was very surprised at this point in the paper to learn about the presence of myoepithelial cells and not only epithelial cells in this system. I think it would be worthy to mention this in the introduction (this is a clear strength of this model system !). If myoepithelial cells are important for organoid - fibroblast interactions, then I imagine

that fibroblasts must interact differently with MCF7 -ras cells that I believe do not have such myoepithelial cells ? (or maybe I missed something ?).

We apologize for not explaining the mammary organoid model in more details. We rectified this in the revised manuscript, where in the Introduction (lines 69-70) we now describe the structure of the organoids consisting of two types of epithelial cells: inner luminal and outer myoepithelial cells, faithfully reflecting the *in vivo* composition of the mammary ducts. Regarding the second point raised, the Reviewer correctly points out that the MCF7-ras spheroid experiments demonstrate that the myoepithelial cells might be dispensable for fibroblast-induced branching because they are absent in these spheroids. We thank the Reviewer for this observation and now discuss this point in the revised manuscript (lines 345-350).

Figure 3D: I am totally confused here ! Can the authors share with me how they identify epithelial cells from myoepithelial cells in EM pictures ? Maybe it is totally obvious to them, but as a naïve reader, I was not even told in the introduction of the paper that this system had myoepithelial cells ! This should be written to be accessible to everybody.
What are the white arrows referring to ? I could not find this info in the legend ?

We apologize for the lack of clarity of Figure 3D. To clarify what is shown in this Figure we include a detailed explanation in the revised legend to Figure 3 legend (lines 898-902). Specifically, identification of mammary epithelial cell types in EM pictures is consistent with published studies (*i.e.* Ewald et al., 2012). In the EM pictures presented, luminal cells are defined as lumen-facing cells with microvilli at their apical membrane and numerous secretory vesicles in their cytoplasm. Myoepithelial cells are in contact with the basement membrane, generally thinner cells with fewer secretory vesicles and organelles in the cytoplasm and with a different electron density of their cytoplasm (darker than the cytoplasm of luminal cells). We are also sorry for forgetting to specify what the arrows refer to in the Figure legend. We have now included it in the revised Figure 3 legend (lines 902-903). The arrowheads point to the layer of extracellular matrix (ECM) that lays between the fibroblasts and myoepithelial cells.

Figure 3F: is this type of quantification really useful ? I think one can see that the cells bind the laminin matrix on the epithelial cells.

We believe that the quantification is useful because it allows a better evaluation of the thin layer of ECM (LAMA5+) between the epithelium and the fibroblasts.

Line 152 "Moreover, the fibroblasts expressed phosphorylated myosin light chain 2 (P-MLC2), a marker of active non-muscle myosin II (Figure 4B)." A cell does not express p-MLC2, it phosphorylates MLC2 !

Sorry for the misunderstanding. The word “express” was here used to signify “show signs of”, not “synthesize protein”. To avoid confusion we have rephrased the sentence to: “Moreover, the fibroblasts stained positively for phosphorylated myosin light chain 2 (P-MLC2)” (lines 167-168).

Figure 4B: yes, there is as expected pMLC2 in epithelia as well as in fibroblasts ! Both cell types display contractile structures !

The Reviewer is correctly pointing that both fibroblasts and epithelial cells display contractile structures. Having a contractile apparatus, however, does not automatically mean actively contracting. Phosphorylation of MLC2, on the other hand, is a marker of active non-muscle myosin II (NMII) and contraction. Therefore, we provide the staining in Figure 4B to show that the fibroblasts in contact with the epithelium are in fact contractile cells.

Figure 4C: it is difficult to interprete this experiment since inhibition of ROCK or MLC will indeed affect both epithelial fibroblast cells.

We agree with the Reviewer that application of small molecule inhibitors to co-cultures will affect both epithelial cells and fibroblasts. That is why (as explained in the text) we had included, as a control, FGF2-induced organoids treated with inhibitors. FGF2-induced organoids produce branches without requiring fibroblasts. Therefore, if their exposure to the tested inhibitors led to branching inhibition, that would indicate that the inhibitors impair epithelial branching by acting on the epithelium. However, inhibition of ROCK or NMII in FGF2-treated organoids did not lead to loss of branching, demonstrating that contractility driven by NMII is dispensable for FGF2-induced branching. Inhibition of ROCK or NMII in fibroblast-organoid co-cultures instead, led to complete loss of organoid branching, demonstrating that fibroblasts contractility is necessary for the epithelial branching. Moreover, we further strengthened the evidence for the requirement of fibroblast contractility for epithelial branching by including experiments with *Myh9*KD/KO fibroblasts, in which we specifically target fibroblast contractility. These experiments demonstrate that fibroblast contractility driven by NMII is necessary for epithelial branching, thus corroborating our findings from experiments using inhibitors.

"178 To test whether epithelial proliferation (and thus expansion) plays a role in organoid branching in
179 co-cultures, we inhibited cell proliferation using aphidicolin (DNA polymerase inhibitor), upon which
180 we observed a severe defect in organoid branching (Figure 5D-F). To test for the possibility that the
181 observed effect could be caused by inhibition of fibroblast proliferation, we performed the experiment
182 also with fibroblasts pretreated with mitomycin C, an irreversible DNA synthesis blocker (Figure 5D).
183 The pretreatment of fibroblast with mitomycin C had no effect on the result (Figure 5D-F),
184 demonstrating that fibroblast proliferation is dispensable while epithelial proliferation is necessary for
185 organoid branching in co-cultures."
I think the authors should really think about their experiments. The perturbations they use will not only induce loss of cell proliferation but also cell death in both cell populations. The epithelium will react with an epithelial homeostasis response. I think the kind of causalities the authors are trying to make are just impossible !

We have obviously considered the possibility of toxicity induced by these drugs and do not quite understand why the Reviewer would think that aphidicolin or mitomycin C are not suitable. Both approaches have been used extensively by the scientific community to inhibit cell proliferation in various systems, including in mammary organoid cultures and explants. For example, aphidicolin has been used to study proliferation-free morphogenesis of optic cup organoids (Eiraku et al. 2011, Okuda et al. 2018) and Huebner and colleagues used aphidicolin to study the mechanisms of epithelial branch elongation. They showed that organoids treated with aphidicolin were able to promote branch elongation, demonstrating that cell proliferation is disposable for branch elongation in mammary organoids (Huebner et al., 2016). In our experiments, we used the same concentrations of aphidicolin and mitomycin C as previously reported by others and we carefully checked the cells and whole organoids for any signs of toxicity. We could not observe increased cell death in the presence of aphidicolin. For clarification, in case of cell death or general suffering, organoids would shed dying cells into the Matrigel and we did not observe dead cells in aphidicolin-treated organoids or co-cultures. We have, however, observed massive cell death in

organoids treated with mitomycin C. That is why we have not used mitomycin C-treated organoids in our study (only fibroblasts were treated with mitomycin C because it was not toxic for them).

Figure 5G: my eyes cannot see the gradient of ERK that the authors see. In panel 5 from the left, I see kind of a gradient of ERK on the left branch of the epithelial bud, but not on the right branch ! The nuclear stain indicates that the right branch is on the right focal plane. It is difficult to conceive how the authors can get a robust statistic with such images. Or then there is a more complex pattern not fully understood ?

Also, work from the 20atsuda lab suggests the existence of discrete ERK pulses, that one could from the image in fig. 5I – P-ERK, in which you can clearly see some ON and OFF cells. So the idea of a gradient of p-ERK signaling is not intuitive.

The analysis of P-ERK was carefully performed, as indicated in the legend to Figure 5, on 19 branches of 10 organoids and reached a strong statistical significance. We agree, however, that the gradient of P-ERK was not continuous and presented a more complex "salt-and-pepper" pattern, with P-ERK high cells predominantly located at branch tips, rather than a gradient. As correctly pointed out by the Reviewer, ERK signaling is very dynamic (Aoki et al., 2017; Blum et al., 2019; Ender et al., 2022; Gagliardi et al., 2021; Hino et al., 2020) and its oscillating component cannot be detected by the analysis of still images. ERK signaling dynamics can be more satisfactorily studied in greater spatiotemporal detail only by live imaging using a live ERK signaling reporter and we believe that such experiments are beyond the scope of this study. Therefore, we decided to exclude the data on P-ERK staining, together with the respective part of the discussion. Instead, we focus only on YAP, a well-known mechanosensor with a much clearer spatial pattern.

References:

Aoki, K., Kondo, Y., Naoki, H., Hiratsuka, T., Itoh, R. E. and Matsuda, M. (2017). Propagating Wave of ERK Activation Orients Collective Cell Migration. *Dev Cell* **43**, 305-317.e5.

Blum, Y., Mikelson, J., Dobrzyński, M., Ryu, H., Jacques, M.-A., Jeon, N. L., Khammash, M. and Pertz, O. (2019). Temporal perturbation of ERK dynamics reveals network architecture of FGF2/MAPK signaling. *Mol Sys Biol* **15**, e8947.

Caruso, M., Huang, S., Mourao, L. and Scheele, C. L. G. J. (2022). A Mammary Organoid Model to Study Branching Morphogenesis. *Front Physiol* **13**, 826107.

Dong, J., Feldmann, G., Huang, J., Wu, S., Zhang, N., Comerford, S. A., Gayyed, M. F., Anders, R. A., Maitra, A. and Pan, D. (2007). Elucidation of a universal size-control mechanism in Drosophila and mammals. *Cell* **130**, 1120–1133.

Dvorak, P., Bednar, D., Vanacek, P., Balek, L., Eiselleova, L., Stepankova, V., Sebestova, E., Kunova Bosakova, M., Konecna, Z., Mazurenko, S., et al. (2018). Computer-assisted engineering of hyperstable fibroblast growth factor 2. *Biotechnol Bioeng* **115**, 850–862.

Ender, P., Gagliardi, P. A., Dobrzyński, M., Frismantiene, A., Dessauges, C., Höhener, T., Jacques, M.-A., Cohen, A. R. and Pertz, O. (2022). Spatiotemporal control of ERK pulse frequency coordinates fate decisions during mammary acinar morphogenesis. *Dev Cell* **57**, 2153-2167.e6.

Ewald, A. J., Huebner, R. J., Palsdottir, H., Lee, J. K., Perez, M. J., Jorgens, D. M., Tauscher, A. N., Cheung, K. J., Werb, Z. and Auer, M. (2012). Mammary collective cell migration involves transient loss of epithelial features and individual cell migration within the epithelium. *J Cell Sci* **125**, 2638–2654.

Gagliardi, P. A., Dobrzyński, M., Jacques, M.-A., Dessauges, C., Ender, P., Blum, Y., Hughes, R. M., Cohen, A. R. and Pertz, O. (2021). Collective ERK/Akt activity waves orchestrate epithelial homeostasis by driving apoptosis-induced survival. *Dev Cell* S1534-5807(21)00436–6.

Gjorevski, N., Nikolaev, M., Brown, T. E., Mitrofanova, O., Brandenberg, N., DelRio, F. W., Yavitt, F. M., Liberali, P., Anseth, K. S. and Lutolf, M. P. (2022). Tissue geometry drives deterministic organoid patterning. *Science* **375**, eaaw9021.

Gouon-Evans, V., Rothenberg, M. E. and Pollard, J. W. (2000). Postnatal mammary gland development requires macrophages and eosinophils. *Development* **127**, 2269–2282.

Gouon-Evans, V., Lin, E. Y. and Pollard, J. W. (2002). Requirement of macrophages and eosinophils and their cytokines/chemokines for mammary gland development. *Breast Cancer Res* **4**, 155–164.

Gyorki, D. E., Asselin-Labat, M.-L., van Rooijen, N., Lindeman, G. J. and Visvader, J. E. (2009). Resident macrophages influence stem cell activity in the mammary gland. *Breast Cancer Res* **11**, R62.

Hino, N., Rossetti, L., Marín-Llauradó, A., Aoki, K., Trepat, X., Matsuda, M. and Hirashima, T. (2020). ERK-Mediated Mechanochemical Waves Direct Collective Cell Polarization. *Dev Cell* **53**, 646-660.e8.

Huang, J., Wu, S., Barrera, J., Matthews, K. and Pan, D. (2005). The Hippo signaling pathway coordinately regulates cell proliferation and apoptosis by inactivating Yorkie, the Drosophila Homolog of YAP. *Cell* **122**, 421–434.

Huebner, R. J., Neumann, N. M. and Ewald, A. J. (2016). Mammary epithelial tubes elongate through MAPK-dependent coordination of cell migration. *Development* **143**, 983–993.

Kasid, A., Lippman, M. E., Papageorge, A. G., Lowy, D. R. and Gelmann, E. P. (1985). Transfection of v-rasH DNA into MCF-7 human breast cancer cells bypasses dependence on estrogen for tumorigenicity. *Science* **228**, 725–728.

Koledova, Z., Sumbal, J., Rabata, A., de La Bourdonnaye, G., Chaloupkova, R., Hrdlickova, B., Damborsky, J. and Stepankova, V. (2019). Fibroblast Growth Factor 2 Protein Stability Provides Decreased Dependence on Heparin for Induction of FGFR Signaling and Alters ERK Signaling Dynamics. *Front Cell Dev Biol* **7**, 331.

Labernadie, A., Kato, T., Brugués, A., Serra-Picamal, X., Derzsi, S., Arwert, E., Weston, A., González-Tarragó, V., Elosegui-Artola, A., Albertazzi, L., et al. (2017). A mechanically active heterotypic E-cadherin/N-cadherin adhesion enables fibroblasts to drive cancer cell invasion. *Nat Cell Biol* **19**, 224–237.

Lilla, J. N. and Werb, Z. (2010). Mast cells contribute to the stromal microenvironment in mammary gland branching morphogenesis. *Dev Biol* **337**, 124–133.

Muzumdar, M. D., Tasic, B., Miyamichi, K., Li, L. and Luo, L. (2007). A global double-fluorescent Cre reporter mouse. *Genesis* **45**, 593–605.

Nguyen-Ngoc, K.-V. and Ewald, A. J. (2013). Mammary ductal elongation and myoepithelial migration are regulated by the composition of the extracellular matrix. *J Microsc* **251**, 212–223.

Ota, M. and Sasaki, H. (2008). Mammalian Tead proteins regulate cell proliferation and contact inhibition as transcriptional mediators of Hippo signaling. *Development* **135**, 4059–4069.

Paine, I. S. and Lewis, M. T. (2017). The Terminal End Bud: the Little Engine that Could. *J Mammary Gland Biol Neoplasia* **22**, 93–108.

Parsa, S., Ramasamy, S. K., De Langhe, S., Gupte, V. V., Haigh, J. J., Medina, D. and Bellusci, S. (2008). Terminal end bud maintenance in mammary gland is dependent upon FGFR2b signaling. *Dev Biol* **317**, 121–131.

Ruta, M., Wolford, R., Dhar, R., Defeo-Jones, D., Ellis, R. W. and Scolnick, E. M. (1986). Nucleotide sequence of the two rat cellular rasH genes. *Mol Cell Biol* **6**, 1706–1710.

Scheele, C. L. G. J., Hannezo, E., Muraro, M. J., Zomer, A., Langedijk, N. S. M., van Oudenaarden, A., Simons, B. D. and van Rheenen, J. (2017). Identity and dynamics of mammary stem cells during branching morphogenesis. *Nature* **542**, 313–317.

Sferruzzi-Perri, A. N., Robertson, S. A. and Dent, L. A. (2003). Interleukin-5 transgene expression and eosinophilia are associated with retarded mammary gland development in mice. *Biol Reprod* **69**, 224–233.

Sumbal, J. and Koledova, Z. (2019). FGF signaling in mammary gland fibroblasts regulates multiple fibroblast functions and mammary epithelial morphogenesis. *Development* **146**, (23):dev185306.

Sumbal, J., Vranova, T. and Koledova, Z. (2020). FGF signaling dynamics regulates epithelial patterning and morphogenesis. *bioRxiv* 2020.11.17.386607.
