# Supplementary material for: Fibroblast-induced mammary epithelial branching depends on fibroblast contractility
Source: PLoS Biol. 2024 Jan 10;22(1):e3002093. doi: 10.1371/journal.pbio.3002093 (PMC10805323; doi:10.1371/journal.pbio.3002093)
Supplement: Attachment — Submitted filename: response to reviewers_231120.pdf [file pbio.3002093.s024.pdf]

Manuscript ID PBIOLOGY-D-23-00673R1

Response to the Editor and Reviewers

Editor's comments:

Data policy:

Many thanks for sending us the data underlying all the graphs shown in the figures. The data files should be saved using the following convention: S1_Data.xlsx (using an underscore).

In addition, please mention in each corresponding figure legend where the data can be found and to name them in the text, please use S1 Data. For example, you can add at the end of each figure legend that contains graphs the following sentence: "The data underlying the graphs shown in the figure can be found in S1 Data."

Done.

Species in abstract:

Please note that per journal policy, the model system/species studied should be clearly stated in the abstract of your manuscript. Please mention that the in vivo experiments have been performed in mice.

The species was included in the abstract as requested. The change was to: "Moreover, we provide evidence for the *in vivo* existence of contractile fibroblasts specifically surrounding terminal end buds of pubertal **murine** mammary glands, advocating for an important role of fibroblast contractility in branching *in vivo*." (lines 31-33). (Please note that line numbers are relevant for the manuscript with track changes in full layout.)

Reviewers' comments:

Reviewer 1:

The authors of this manuscript have made substantial efforts to improve the manuscript and provide a wealth of additional data that substantially strengthens the paper.

Here I will comment the responses provided to my main criticism:

1. In vivo importance of the observed phenomenon.

To address this issue, the authors now provide 3D images of pubertal glands showing that the necs of growing TEBs are associated with contractile (aSMA+) fibroblasts and that bifurcating tips are associated with "looping" fibroblasts. While the images provided are gorgeous, and undoubtedly motivate the authors to continue their research toward in vivo analyses and approaches, without quantifications these new images remain anecdotal (at which point of TEB bifurcation they appear, are they associated with all bifurcating TEBs, is there truly a loop - the video of confocal stacks suggests that the loop is only partial, not extending the entire circumference of the nascent new tip etc.).

It is fully understandable that within the revision timeframe obtaining enough material for quantification may not be feasible, but this also means that the authors have to remain cautious about their conclusions. This works mostly throughout the manuscript, yet e.g. the very last concluding chapter of the Discussion includes an overinterpretation: "In conclusion, we find that fibroblasts drive branching morphogenesis of the mammary gland by exerting mechanical forces on epithelial cells." This is a fair conclusion on the organoid data, but the new in vivo images are

not sufficient to justify this conclusion on the pubertal mammary gland, but obvious it is a good basis for a hypothesis that this might be the case.

I also think that the authors should discuss their fibroblast looping model in light of the recent paper from the Ewald lab (PMID: 36602106) showing that genuine tip bifurcations can occur also in the classic organoid set-up, i.e. without fibroblasts (while to me it still remains unclear to which extent in the organoids - with or without fibroblasts - branching really recapitulates true bifurcation. Or are majority of the branching events in fact 'bulging/blebbing' of the bilayered stalk once it has proliferated sufficiently to have enough cells for a new branch?). Similarly, another group of has shown that in embryonic mammary organoids, bifurcations may occur in the absence of stromal cells (PMID: 37367826).

We agree with the Reviewer that the data collected during the timeframe of the revision are not sufficient to provide a robust quantification of the fibroblast localization at TEBs during ductal growth and bifurcation and we agree that quantitative volumetric imaging of the TEBs within their microenvironment should be further used to better understand the branching morphogenesis of pubertal mammary gland. To avoid overinterpreting the data, we have toned down the last part of discussion (lines 399-402) to: "In conclusion, we find that fibroblasts drive branching morphogenesis in mammary organoids by exerting mechanical forces on epithelial cells. These observations support the hypothesis that contractile fibroblasts drive pubertal mammary branching; however, future *in vivo* studies will be needed to formally demonstrate this."

We have added the information about organoid branch bifurcation in the absence of fibroblasts to the discussion session (line 367), where we discuss how paracrine growth factors are used to induce organoid branching in stromal cell-free culture: "…can induce organoid branching in the absence of fibroblasts when added to the medium in nanomolar concentrations (Sternlicht et al., 2005), including bifurcation of the organoid branches (Neumann et al. 2023)."

In the paper by Myllimaki et al. (PMID: 37367826), the authors used a very different culture method for most of their experiments: tissue explants of embryonic mammary glands that contain both the mammary epithelium and surrounding mesenchymal cells including fibroblasts. The embryonic mammary epithelium was grown in Matrigel, in the absence of stromal cells only in one experiment (Figure 4M); however, human FGF2 was added to the culture medium to induce organoid budding and no clear bifurcation events were visible in these conditions. Therefore, this work is not discussed in this context.

We agree, however, that the extent to which the available organoid models reflect the *in vivo* situation of mammary branching is still unclear and future studies that will include intravital imaging will be vital to unravel the modalities and mechanisms of mammary branching morphogenesis.

2. Is the organoid model a good model of TEBs due to the different cellular architecture (TEBs being multilayered, organoids bilayered)?

The authors now provide new data with FGF2-STAB + fibroblasts and show that this leads to a more multicellular structure and still the fibroblasts appear to loop the same way and lead to faster onset of budding/branching that FGF2-STAB alone (which also induces budding/branching, but apparently only after 4 days of culture). This is a nice addition to the paper but does not exclude the fact that majority of the fibroblast data is from bilayered organoids. Again, I call after caution in data interpretation.

Yes, most of the data is from bi-layered structures – we wanted to keep the co-cultures free of added paracrine factors in order to distinguish the effects of paracrine signaling from the effect of fibroblast contractility.

To avoid overinterpreting our findings, we changed the summarizing part of the Result section to: “These results demonstrate that a combination of contractile fibroblasts and strong proliferative signals can reproduce several aspects typical for TEB branching in organoid cultures.” (lines 255-257).

3. Somewhat scarce quantification of a number of different data in the original version of the manuscript.

The authors are to be applauded for their work on fixing this issue - the manuscript is much stronger now than it was.ns

We thank the Reviewer for their appreciation of our work and we fully agree that the revisions have considerably increased the quality and significance of our study.

4. The question on contractility vs. motility

This question has been addressed appropriately. The finding that fibroblasts may also play a role branch stabilization is a nice addition to the manuscript.

We thank the Reviewer for their recognition of the importance of the newly added data.

5. Importance of patterned cell proliferation in organoids.

The authors give a plausible explanation for the difference between organoids and TEBs, but again, this is one additional difference between them, further warranting caution in overinterpreting the in vivo relevance of the organoid data.

To avoid overinterpreting the data, we have tuned down the discussion of *in vivo* relevance of the organoid data.

6. YAP

I remain confused about YAP and its potential link with the contractile fibroblasts.

The authors write" …indicating that YAP signal arises from the contact with contractile fibroblasts and not the overall shape of the epithelial branch" and "Knockout of Myh9 in fibroblasts prevented YAP pattern formation (Figure 4J), indicating that contact with contractile fibroblasts regulates YAP epithelial distribution."

Am I interpreting Fig. 4J correctly? To me it looks that in organoids with Myh9 deleted fibroblasts, YAP is high in all cells. So how can "YAP signal arise from the contact with contractile fibroblasts"?

I think this is the weakest part of the manuscript, and I recommend the authors to consider removing it from the manuscript. Previous (PMID: 24589775) and recent (https://www.biorxiv.org/content/10.1101/2023.08.23.554465v1.full) reports indicate that YAP is highest in growing TEBs, which according to the current manuscript are largely devoid of fibroblasts (except upon bifurcation).

So, to me the correlation between YAP and proliferation appears much stronger than between YAP and contractile fibroblasts.

We concur with the Reviewer that experiment in Figure 4J is confusing. The way we understand the outcome is that fibroblast contractility is important to establish the observed pattern of YAP signal in branched organoid (nuclear YAP in the branch stalks, cytoplasmic YAP at the tips), however, nuclear YAP is lost when a similar shape is established by addition of exogenous FGF2 in the medium (Figure 4 G-I), suggesting an important link between the presence of fibroblasts and YAP signaling. When we knocked out *Myh9*, organoids did not form budding structures and all their cells presented nuclear YAP within spherical organoids. This may be partially linked to proliferation: organoids co-cultured with *Myh9*-KO fibroblasts are still receiving paracrine signals from the fibroblasts and proliferate. Indeed, organoids co-cultured with Myh9-KO fibroblasts are often stratified, suggesting active proliferation. Furthermore, the proliferation data presented in Supplementary Figure 10 indicate a positive correlation between the number of proliferative cells and YAP nuclear localization.

To reflect the intricate complexity of interpretation of these data, we have rephrased the part of Results related to Figure 4G-J (lines 206-212): "…indicating that YAP activation in epithelial cells at the neck of elongating buds is induced by the contact with contractile fibroblasts and not simply by the overall shape of the epithelial bud. However, knockout of *Myh9* in fibroblasts resulted in round organoids with no branching and a homogeneous distribution of nuclear YAP (Figure 4J). Our results show that while fibroblast contractility is necessary for the formation of branch with patterned YAP signal, the nuclear translocation of YAP can happen even in the absence of fibroblast contractility."

And we have added a paragraph in the Discussion related to YAP activation in epithelial cells (lines 377-380): "It remains unclear though whether fibroblasts induce YAP activation to promote epithelial proliferation at the neck and thus bud elongation, or if the patterned YAP activation in epithelial buds reflects the proliferative status of cells located in different regions of the organoid."

7. One final point about Discussion. There, the authors speculate that "in vivo the highly dynamic mechanically active fibroblasts could initiate formation of epithelial clefts and further reinforce them by subsequent deposition and remodeling of ECM." I wonder if the authors would like to ponder this speculation in light of the evidence indicating that pubertal branching morphogenesis is highly stochastic, following "a branching and annihilating random walk" model (PMID: 28135720).

We thank the Reviewer for this suggestion; accordingly, we have added the following sentence to the discussion session (lines 335-338): "Recently published simulations suggest that mammary pubertal branching is highly stochastic, however its overall shape depends on the angle of TEB bifurcation (Hannezo et al. 2017; Nerger et al., 2021). Thus, the local effect of fibroblast contractility could affect the random branching pattern."

Reviewer 2:

The authors have performed a lot of experiments to satisfy my concerns, as well as those of the other reviewer. Most importantly, they have clarified a lot of points that make the manuscript more accessible. I think it was a good decision to leave out the MAPK dataset that is difficult to interpret. I think that the paper is a good example of next generation organoid in which tackles how multiple cell types contribute to organogenesis. I am happy to accept the manuscript, and congratulate the authors.

We thank the Reviewer for insightful comments that helped to improve the quality of the manuscript.
